# Melittin- and Apamin-Standardized Bee Venom: UHPLC-ESI-MS Characterization, Antibacterial Activity, and Apoptosis Induction in Human Keratinocytes

**DOI:** 10.3390/molecules31162787

**Published:** 2026-08-11

**Authors:** Anna Kurek-Górecka, Małgorzata Kłósek, Dagmara Jaworska, Anna Mertas, Radosław Balwierz, Sebastian Granica, Mirosław Szczepański, Katarzyna Piszczatowska, Sevgi Kolayli, Zenon P. Czuba

**Affiliations:** 1 Department of Microbiology and Immunology, Faculty of Medical Sciences in Zabrze, Medical University of Silesia in Katowice, Jordana 19, 41-808 Zabrze, Poland; mklosek@sum.edu.pl (M.K.); djaworska@sum.edu.pl (D.J.); amertas@sum.edu.pl (A.M.); zczuba@sum.edu.pl (Z.P.C.); 2 Institute of Chemistry, University of Opole, Oleska 48, 45-052 Opole, Poland; radoslaw.balwierz@uni.opole.pl; 3 Department of Pharmaceutical Biology, Medical University of Warsaw, 1 Banacha St., 02-097 Warsaw, Poland; 4 Department of Biochemistry, Medical University of Warsaw, 02-091 Warsaw, Poland; mszczepanski@wum.edu.pl (M.S.); katarzyna.piszczatowska@wum.edu.pl (K.P.); 5 Department of Chemistry, Faculty of Science, Karadeniz Technical University, 61080 Trabzon, Türkiye; skolayli@ktu.edu.tr; 6 Faculty of Medicine, Nakhchivan State University, Nakhchivan AZ7012, Azerbaijan

**Keywords:** bee venom, melittin, apamin, antibacterial agent, skin

## Abstract

Bee venom (BV) is valued therapeutically mainly for its peptides, melittin and apamin. This study determined the dose-dependent biological activity of bee venom standardized to its melittin and apamin content by UHPLC-ESI-MS, as well as that of its principal peptides, melittin and apamin, tested at matched concentrations. Antibacterial activity was assessed as showing the minimum inhibitory and minimum bactericidal concentrations (MIC, MBC) against *Staphylococcus aureus* (MSSA and MRSA) and *Staphylococcus epidermidis* (MRSE). Cytotoxicity toward primary human keratinocytes (HEKa) was determined by MTT and LDH assays and the mode of cell death by Annexin V-FITC/propidium iodide staining. The standardized bee venom contained 608.21 µg/mg of melittin and 22.17 µg/mg of apamin. Melittin was the only active constituent (MIC 2.5 to 5 µg/mL, MBC/MIC ≤ 2), whereas apamin was inactive (>10 µg/mL). Bee venom was active only against *Staphylococcus epidermidis* (MRSE), with both MIC and MBC values equal to 10 µg/mL. At concentrations of 5 and 10 µg/mL, both bee venom and melittin were markedly cytotoxic to keratinocytes. The selectivity index (CC50/MIC) did not exceed 1.6 for melittin and reached 0.2 for bee venom. On a melittin-equivalent basis, whole venom was more cytotoxic than pure melittin (CC50 1.33 versus 3.91 µg/mL), indicating that constituents other than melittin contribute to keratinocyte damage. Melittin is therefore the principal antibacterial constituent, but its narrow selectivity does not support direct topical use. Combination experiments evaluating bee venom or its isolated peptides in combination with antibiotics were not performed here and remain a defined direction for future work. Standardization to melittin content predicts potency but not safety, which has to be assessed on the whole standardized venom.

## 1. Introduction

Bee venom (BV), also known as apitoxin, is secreted by the poison glands of honeybee workers. Each honeybee sting delivers approximately 50–140 μg of BV, composed of a complex mixture of biologically active compounds [1]. Although it is used as a defense mechanism, its complex composition, rich in biologically active peptides, enzymes and biogenic amines, suggests considerable therapeutic potential, especially for dermatological and topical applications. Experimental studies and clinical reports have proven that BV exhibits anti-inflammatory, anti-apoptotic, anti-fibrotic, antibacterial, antiviral, antifungal, and even anticancer activity [2,3,4,5,6,7,8]. Due to its anti-inflammatory and antibacterial properties, bee venom has been considered for therapeutic applications in skin diseases [3].

Bee venom consists of approximately 80% water. Melittin (MEL) is the major bioactive peptide of bee venom and constitutes approximately 40–60% of the venom dry mass. It is a small 26-residue amphipathic peptide that adopts a bent α-helical conformation, with a predominantly hydrophobic N-terminal region and a positively charged C-terminal segment, and this amphipathicity underlies its ability to insert into and permeabilize lipid membranes. Among the various bioactive constituents of BV are phospholipase A2 (PLA2; approximately 12% of the dry weight of BV), mast cell degranulating peptide (MCDP; approximately 3% of the dry weight of BV), apamin (APA; approximately 2–3% of the dry weight of BV), secapin (approximately 0.5% of the dry weight of BV), adolapin (approximately 1% of the dry weight of BV), tertiapin (approximately 0.1% of the dry weight of BV), hyaluronidases (approximately 1.5–2% of the dry weight of BV), and acid phosphatase (approximately 1% of the dry weight of BV). Other peptides constitute less than 15% of the dry mass of BV. Among them are dipeptidyl peptidase IV, which is also known as allergen C (Api m 5); serine protease inhibitor (Api m 6); CUB serine protease (Api m 7); icarapin (Api m 10); and major royal jelly proteins (MRJP 8 and MRJP 9). In addition to peptides and proteins, bee venom contains a variety of low-molecular-weight compounds, including biogenic amines (histamine, dopamine, and noradrenaline), carbohydrates (mainly glucose and fructose), amino acids, phospholipids, trace bioelements such as phosphorus, sulfur, potassium, copper, zinc, sodium, magnesium, calcium, and boron, as well as volatile compounds including pheromones [6,9,10].

Bee venom was standardized to its melittin and apamin content because these two peptides account for its principal biological activities. Melittin possesses anti-inflammatory activity through toll-like receptor (TLR 2 and TLR 4) blockade and inhibition of Platelet-Derived Growth Factor (PDGF) and nuclear factor kappa-light-chain enhancer of activated B cells (NF-ĸB). Melittin is responsible for the antibacterial activity of BV because it interacts with the bacterial cell membrane, leading to disruption of membrane integrity, increased membrane permeability, cellular deformation, and leakage of cytoplasmic contents. Gram-positive bacteria appear to be more susceptible to melittin due to easier penetration through the peptidoglycan layer, whereas Gram-negative bacteria possess an additional lipopolysaccharide outer membrane barrier. Moreover, a proline residue has been demonstrated to play a crucial role in the antimicrobial activity of melittin [11].

Its antimicrobial effects have been demonstrated against *Staphylococcus aureus* (including MRSA and MSSA strains), *Escherichia coli*, *Pseudomonas aeruginosa*, *Acinetobacter baumannii*, *Listeria monocytogenes*, *Borrelia burgdorferi*, *Klebsiella pneumoniae*, *Bacillus subtilis*, *Enterococcus faecalis*, *Streptococcus mutans*, *Streptococcus salivarius*, *Streptococcus sobrinus*, *Streptococcus sanguinis*, *Streptococcus mitis*, and *Lactobacillus casei* [12,13].

Apamin exhibits weaker direct antibacterial activity compared to melittin; however, it contributes to the antimicrobial and anti-inflammatory properties of bee venom. Studies demonstrated that apamin inhibited inflammatory responses induced by *Alternaria alternata* and *Aspergillus fumigatus* through suppression of interleukin-6 (IL-6) and interleukin-8 (IL-8) production, as well as modulation of the main signal transducer Smad2/3 and p38 mitogen-activated protein kinase (MAPK) signaling pathways [12,14].

Although melittin is considered the principal bioactive component of bee venom, it remains unclear to what extent the biological activity of whole bee venom reflects the effects of its selected peptides alone. The novelty of this study lies in the direct comparison of standardized bee venom with its two peptides, melittin and apamin, tested at identical concentrations. This approach enabled assessment of whether the biological activity of whole bee venom can be explained solely by its principal peptides or whether additional bee venom constituents contribute to its overall effects. Because bee venom is increasingly considered for topical dermatological applications, Primary Epidermal Keratinocytes, Normal Human Adult (HEKa), were selected as the experimental model to assess its local safety profile. Cytotoxicity was evaluated using complementary assays addressing different aspects of cell damage. The MTT assay was used to assess cellular metabolic activity and viability, the LDH assay to determine plasma membrane disruption associated with lytic cytotoxicity, and Annexin V/PI staining to distinguish apoptotic and necrotic cell death. The combined use of these methods enabled a comprehensive characterization of the effects of standardized bee venom and its major peptides on epidermal cells relevant to potential topical application. Human keratinocytes were selected because they represent the primary cellular target following topical application of bee-venom-containing formulations. Evaluating both antibacterial efficacy and keratinocyte safety within the same experimental framework allows estimation of the therapeutic window relevant to potential dermatological use. The design of the study was fixed in advance around three elements: first, a bacterial panel deliberately restricted to staphylococci, for the reasons set out in the Discussion; second, a two-tier dosing scheme in which whole venom and the isolated peptides were compared both at equal total concentrations and at equal melittin content, the latter being possible only because the venom had been quantitatively standardized; and third, a pre-specified integrative endpoint, the selectivity index (SI = CC50/MIC), calculated from antibacterial and cytotoxicity data generated on the same standardized material and under the same conditions.

## 2. Results

### 2.1. Determination of Melittin and Apamin Content in a Bee Venom Sample by UHPLC-ESI-MS Analysis

UHPLC-ESI-MS analysis of the bee venom sample revealed a high content of melittin equal to 608.21 ± 35.16 µg/mg of bee venom, which accounted for (60.82 ± 3.52)% of the total composition. The content of apamin was equal to 22.17 ± 1.73 µg/mg of bee venom, which accounted for (2.22 ± 0.17)% of the total composition (Table S1, Figures S1–S6). The precision of the analytical method, expressed as the coefficient of variation (CV), was 5.78% for melittin and 7.82% for apamin. The obtained results indicated that melittin is the main component of BV and may be used for standardization of BV. Melittin, as the predominant bioactive peptide of bee venom, and apamin, as another pharmacologically active constituent, may be considered suitable marker compounds for bee venom standardization [3,8]. Owing to its significant abundance, well-characterized structure, and documented biological activity, melittin content may serve as a reliable parameter for assessing the quality, reproducibility, and biological consistency of bee venom preparations. Furthermore, quantitative determination of melittin and apamin could contribute to improving the comparability and safety of bee venom intended for biomedical and pharmaceutical applications.

### 2.2. Cell Viability Assessed by MTT Assay

**Figure 1:**
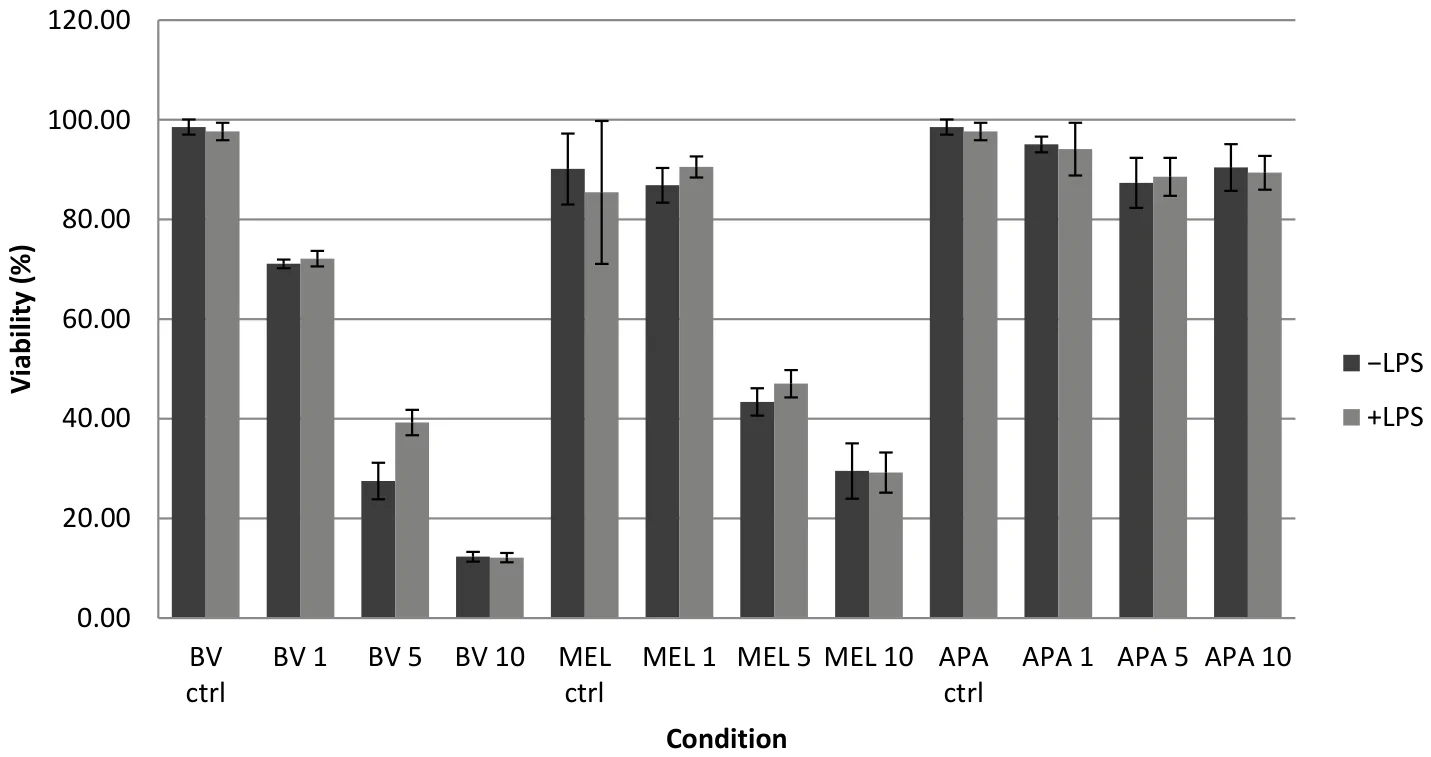
Viability of HEKa cells treated with BV, MEL and APA without or with lipopolysaccharide (LPS) co-stimulation evaluated by the MTT assay (*n* = 4). BV-bee venom, MEL-melittin, APA-apamin. ctrl denotes the vehicle control assayed on the same plate as the corresponding compound. Bee venom and apamin were assayed on one plate and melittin on a separate plate, and each compound is referenced to the vehicle control of its own plate. Bars show mean ± SD.

The viability of the HEKa cell line after the application of BV, MEL and APA was estimated using the MTT assay (3-[4,5-dimethylthiazol-2-yl]-2,5-diphenyltetrazolium bromide). BV, MEL and APA were tested at three concentrations (10, 5 and 1 µg/mL), each under two conditions, without bacterial lipopolysaccharide (−LPS) and with LPS co-stimulation (+LPS). The +LPS condition was included to model an inflammatory, infection-like microenvironment of the skin and to test whether such stimulation modifies the cytotoxic response. The results are presented in Figure 1.

Cell viability is expressed as a percentage of the vehicle control assayed on the same plate, shown as the ctrl columns in Figure 1. The vehicle control reached (98.5 ± 1.5)% without LPS and (97.7 ± 1.8)% under LPS co-stimulation for bee venom and apamin, confirming that the vehicle itself did not appreciably affect viability. Against this reference, the greatest reduction was observed for bee venom and melittin at 10 µg/mL under both conditions. Bee venom at 10 µg/mL reduced viability to (12.3 ± 1.0)% without LPS and to (12.1 ± 0.9)% with LPS co-stimulation (Figure 1). Similarly to BV, melittin at the concentration of 10 μg/mL decreased cell viability from (90.1 ± 7.1)% to (29.5 ± 5.6)% and after LPS stimulation from (85.4 ± 14.3)% to (29.2 ± 4.0)%, respectively (Figure 1).

Also, at the lower concentration of 5 µg/mL, we observed a pronounced decrease in viability for BV and MEL. For BV, cell viability decreased from (98.5 ± 1.5)% to (27.5 ± 3.7)% and after LPS stimulation from (97.7 ± 1.8)% to (39.2 ± 2.5)%, respectively (Figure 1). For melittin, cell viability decreased from (90.1 ± 7.1)% to (43.4 ± 2.7)%, and after LPS stimulation from (85.4 ± 14.3)% to (47.0 ± 2.7)%, respectively (Figure 1). At the lowest concentration of BV and melittin, the cell viability remained above 70%. Therefore, according to ISO 10993-5:2009 criteria, they are classified as non-cytotoxic [15].

No cytotoxic effect was observed following treatment with apamin. Cell viability after exposure to apamin at all three tested concentrations (10, 5 and 1 µg/mL) remained above 70%. The greatest reduction in cell viability was observed at a concentration of 5 µg/mL, where cell survival reached (87.3 ± 5.0)% in non-stimulated cells and (88.6 ± 3.8)% following LPS stimulation, indicating the absence of significant cytotoxicity under the tested experimental conditions (Figure 1).

### 2.3. Lactate Dehydrogenase (LDH) Release Assay

**Figure 2:**
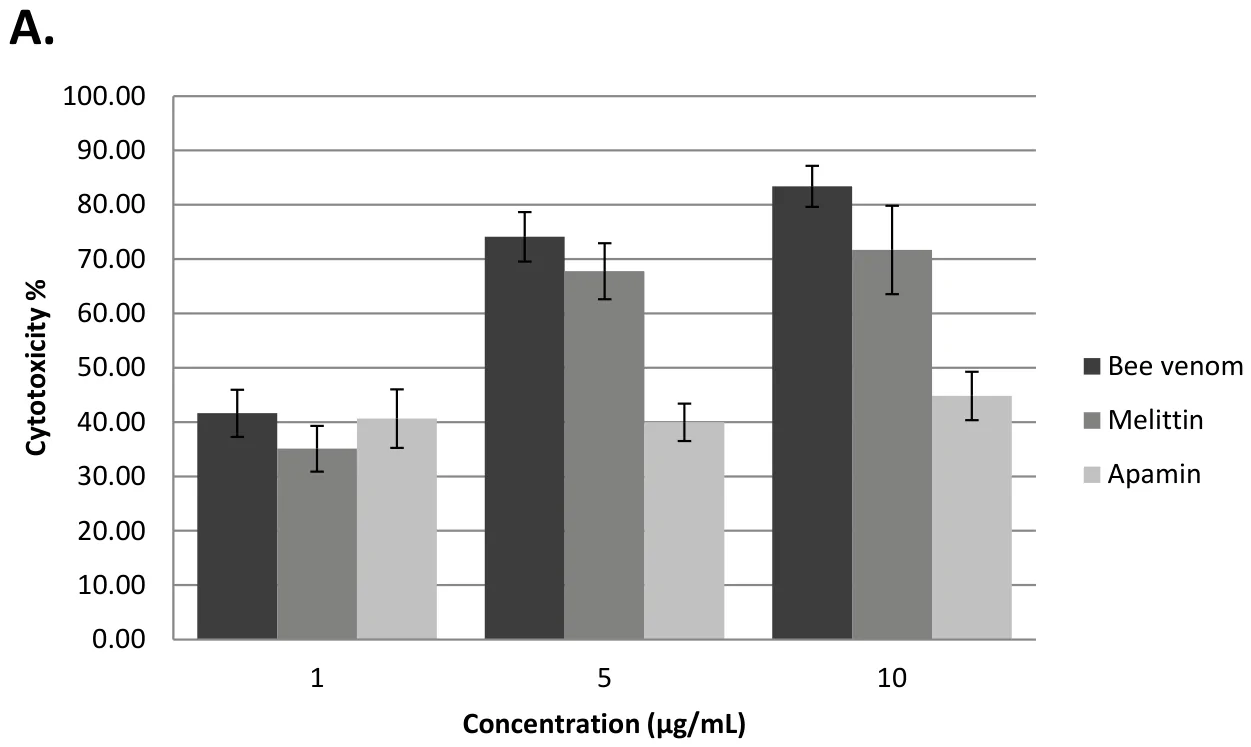

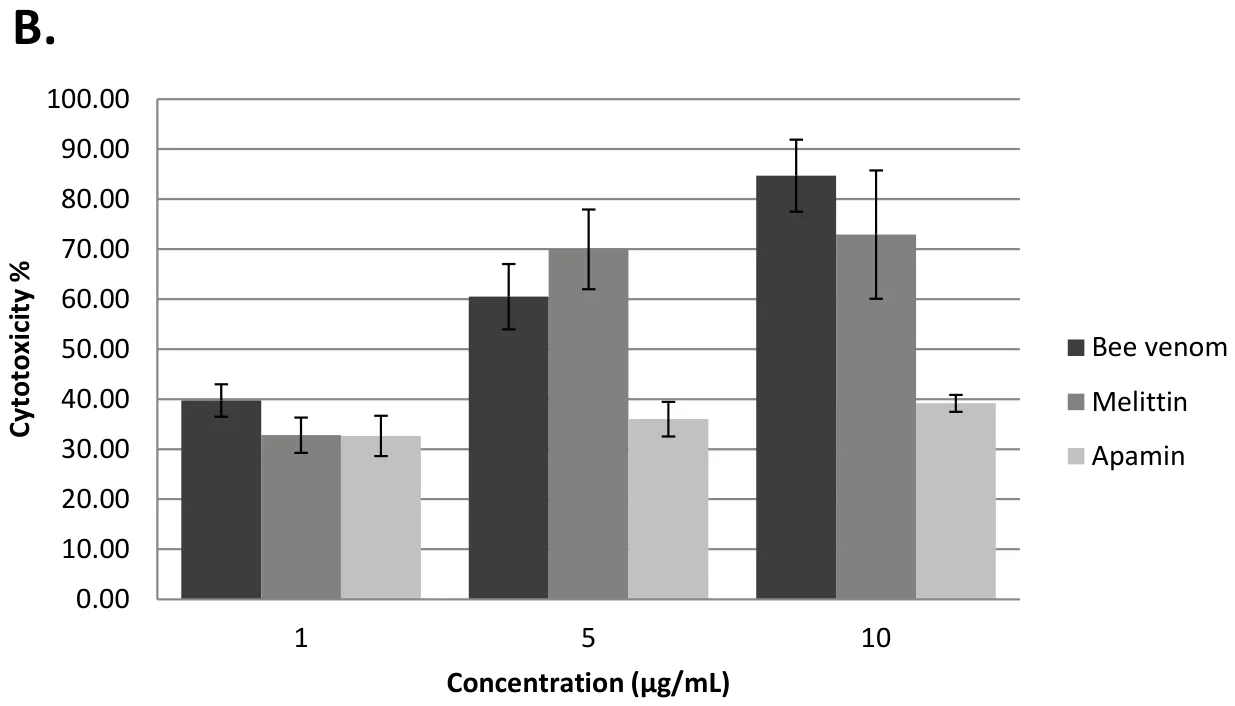
Percentage cytotoxicity in HEKa keratinocytes exposed for 24 h to bee venom (BV), melittin (MEL) and apamin (APA) at concentrations of 1, 5 and 10 µg/mL, without (**A**) or with (**B**) lipopolysaccharide (LPS) co-stimulation, determined by the LDH release assay. Cytotoxicity is expressed relative to the maximum LDH release obtained with 2% Triton X-100. ctrl denotes the DMSO vehicle control assayed on the same plate as the corresponding compound. Bars show mean ± SD (*n* = 4). The underlying per-well values are given in Table S13 and the statistical comparisons in Tables S9–S11.

In the LDH cytotoxicity assay, based on the detection of extracellularly released lactate dehydrogenase from damaged cells, the highest cytotoxicity of BV was observed at concentrations of 10 µg/mL and 5 µg/mL, both in unstimulated cells and following LPS stimulation (Figure 2A,B). The cytotoxicity determined for bee venom at 10 µg/mL was (83.4 ± 3.8)% in non-stimulated cells (Figure 2A) and (84.7 ± 7.2)% following LPS stimulation (Figure 2B). This difference was not statistically significant (Welch *t*-test with Holm correction, Table S10), indicating that LPS co-stimulation did not further increase membrane damage at this concentration. BV at a concentration of 5 µg/mL exhibited cytotoxicity of (74.1 ± 4.6)% in non-stimulated cells and (60.5 ± 6.5)% following LPS stimulation. A similarly high cytotoxic effect was also documented for melittin at concentrations of 10 µg/mL and 5 µg/mL (Figure 2A,B). Melittin at the concentration of 10 µg/mL exhibited cytotoxicity of (71.7 ± 8.1)% in non-stimulated cells and (72.9 ± 12.8)% following LPS stimulation. Similarly, at the lower concentration of 5 µg/mL, melittin also exhibited high cytotoxicity of (67.8 ± 5.1)% in non-stimulated cells and (70.0 ± 8.0)% following LPS stimulation. In contrast, apamin exhibited the lowest cytotoxicity. Apamin at a concentration of 10 µg/mL exhibited a cytotoxicity of (44.8 ± 4.5)% in non-stimulated cells (Figure 2A), which, although the lowest among the tested compounds, remained above the vehicle control and should not be interpreted as an absence of membrane effect. These findings indicate that BV and melittin exerted a more pronounced membrane-damaging effect compared to apamin under the tested experimental conditions.

### 2.4. Effects of Bee Venom and Its Peptides on Apoptosis Assessed by FITC Annexin V Staining 

**Figure 3:**
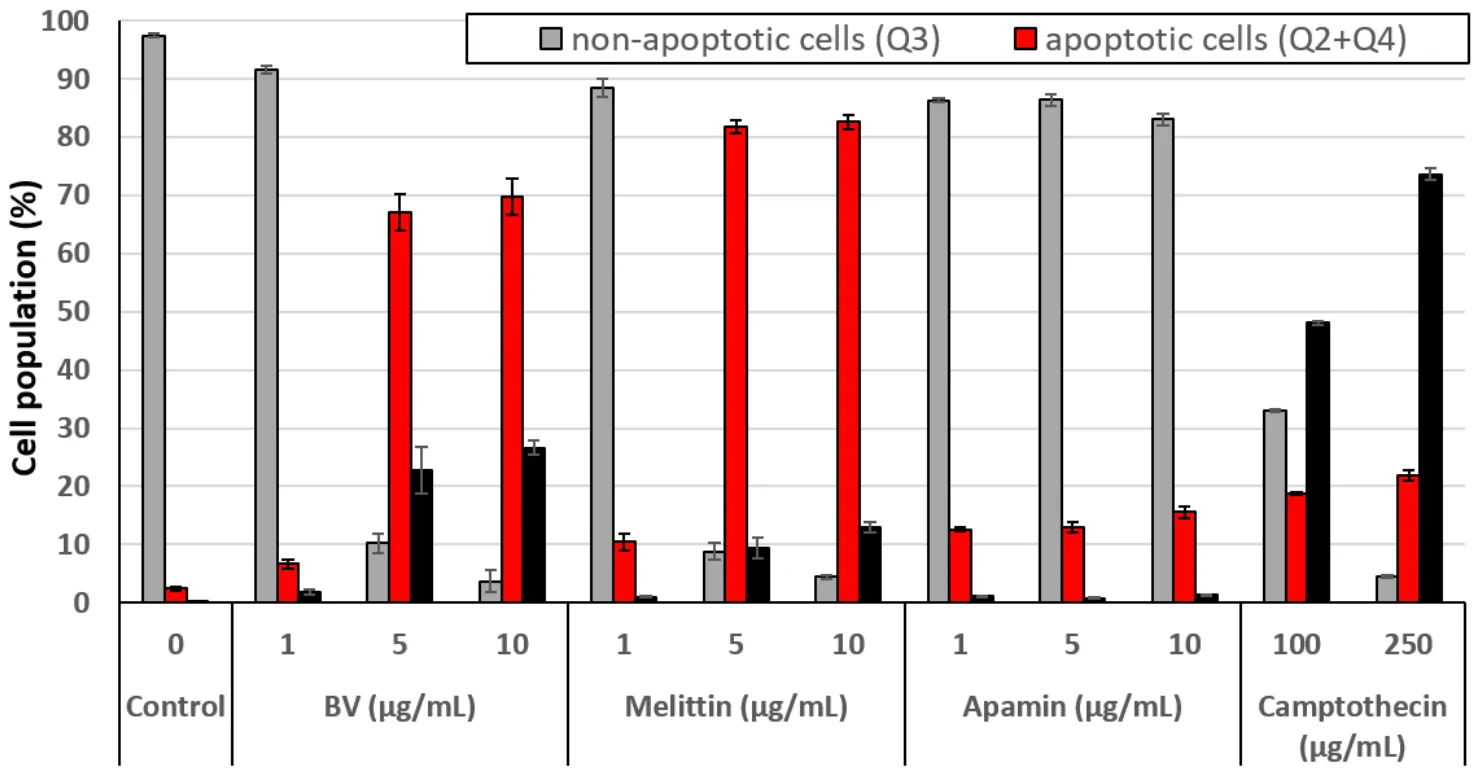
Apoptotic effect of bee venom (BV), melittin and apamin (1, 5 and 10 µg/mL) and camptothecin (100 and 250 µM, positive control) in HEKa cells after 24 h, determined by Annexin V-FITC/propidium iodide staining and flow cytometry. Total apoptosis is the sum of the early (Annexin V+/PI−) and late (Annexin V+/PI+) fractions. The values represent mean ± SD (*n* = 3). Black bars indicate dead cells. The full quadrant distribution is given in Table S5 and representative dot plots in Figure S11.

Our study showed that bee venom and its main component melittin induced apoptosis in HEKa cells in a concentration-dependent manner (Figure 3). In our study, we used BV, melittin and apamin in concentrations of 10, 5 and 1 μg/mL. At a concentration of 10 μg/mL, BV induced apoptosis in (69.7 ± 3.08)% of HEKa cells, and at a concentration of 5 μg/mL, it induced apoptosis in (67.1 ± 3.13)%. At a concentration of 1 μg/mL, BV induced apoptosis in only (6.63 ± 0.75)% of HEKa cells. Accordingly, the percentage of necrotic cells was (26.63 ± 1.2)% for 10 μg/mL BV, (22.7 ± 4.05)% for 5 μg/mL BV, and (1.8 ± 0.35)% for 1 μg/mL BV. Melittin, used as a pure compound, at a concentration of 10 μg/mL induced apoptosis in (82.63 ± 1.25)% of HEKa cells, and at a concentration of 5 μg/mL, induced apoptosis in (81.77 ± 1.08)%. At a concentration of 1 μg/mL, melittin induced apoptosis in (10.43 ± 1.52)% of HEKa cells. The percentage of necrotic cells was (12.93 ± 0.90)% for 10 μg/mL melittin, (9.4 ± 1.83)% for 5 μg/mL, and (1.0 ± 0.17)% for 1 μg/mL.

In contrast to melittin, apamin, the other constituent of bee venom studied here, showed no pro-apoptotic activity. Apamin, used as a pure compound, at a concentration of 10 μg/mL induced apoptosis in only (15.6 ± 1.01)% of HEKa cells; at a concentration of 5 μg/mL, it induced apoptosis in (12.87 ± 0.90)%; and at a concentration of 1 μg/mL, it induced apoptosis in (12.60 ± 0.30)%. The percentage of necrotic cells was (1.30 ± 0.10)% for 10 μg/mL apamin, (0.77 ± 0.06)% for 5 μg/mL, and (1.07 ± 0.15)% for 1 μg/mL. The results described above show that the main pro-apoptotic effect of BV is due to the presence of melittin. The results are presented in Figure 3 below.

### 2.5. Determination of the Minimum Inhibitory Concentration (MIC) and Minimum Bactericidal Concentration (MBC) of Bee Venom, Melittin and Apamin

In the microbiological assays, melittin was identified as the only biologically active component of bee venom, exhibiting antibacterial activity with MIC values of 2.5–5 µg/mL and an MBC/MIC ratio ≤ 2, indicating a bactericidal effect (Table 1). The lowest MIC value was observed against *Staphylococcus epidermidis* MRSE, suggesting the highest susceptibility of this strain to melittin (Table 1). In contrast, apamin showed no antibacterial activity at the tested concentrations (>10 µg/mL), whereas bee venom demonstrated activity exclusively against methicillin-resistant *Staphylococcus epidermidis* MRSE. Chlorhexidine solution (CHX) used as a positive control exhibited greater antimicrobial efficacy against *Staphylococcus epidermidis* MRSE and *Staphylococcus aureus* MSSA than bee venom and its isolated components, including melittin and apamin. However, in the case of the tested *Staphylococcus aureus* MRSA strain, CHX exhibited a higher MBC value compared with melittin (Table 1).

### 2.6. Correlation Analysis Between Bee Venom Composition and Its Cytotoxic, Pro-Apoptotic, and Antibacterial Properties

The results obtained from the different assays were mutually consistent. Loss of cell viability was associated with membrane damage (Spearman’s ρ = −0.80; *p* = 0.010), while apoptosis induction correlated with increased LDH release (ρ = +0.75; *p* = 0.020); however, the relationship between viability and apoptosis did not reach statistical significance (ρ = −0.58; *p* = 0.099). These findings indicate a consistent direction of cellular responses under the investigated experimental conditions.

**Figure 4:**
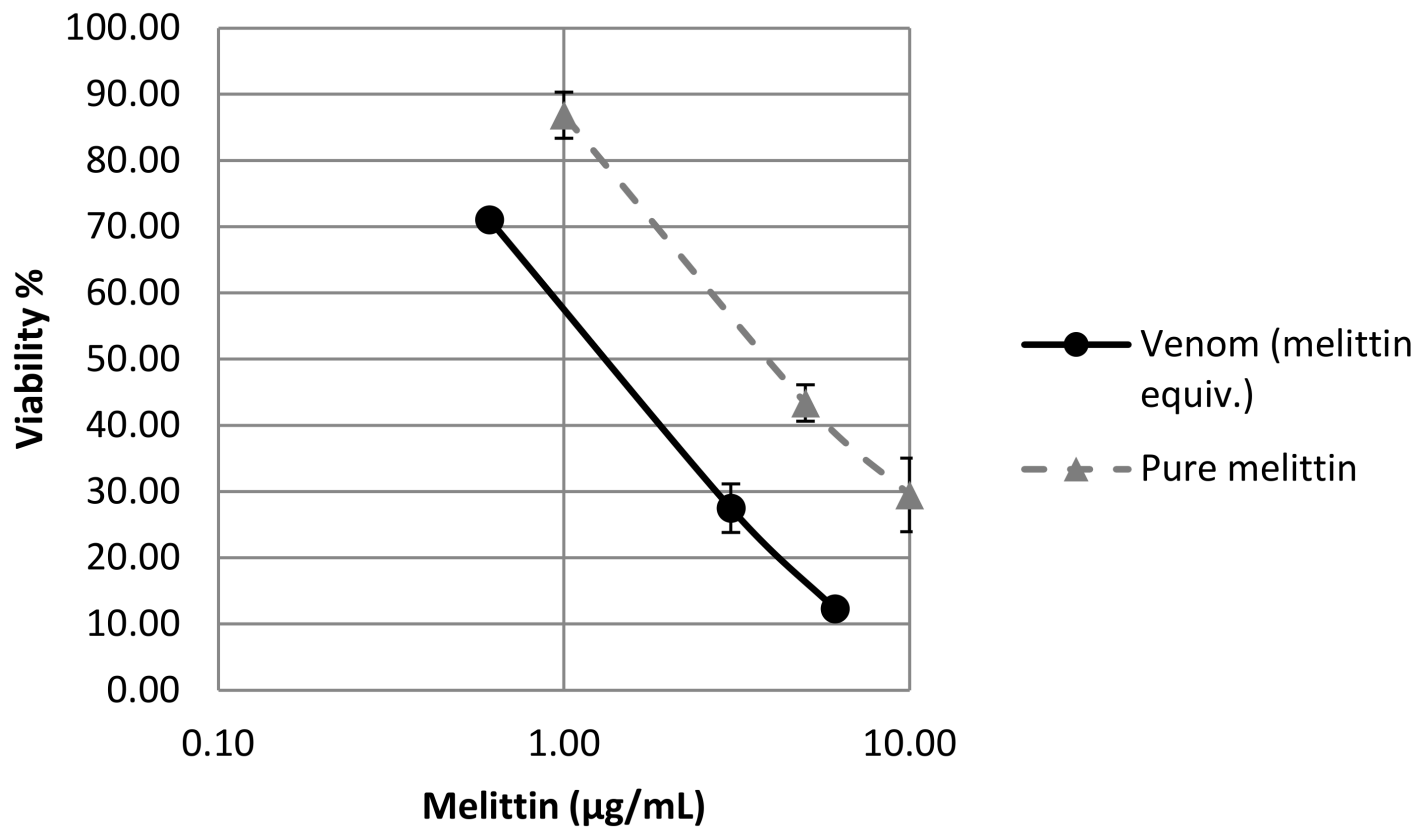
Dose-dependent effects of bee venom (expressed as melittin-equivalent concentration) and pure melittin on HEKa cell viability determined by the MTT assay. Bee venom concentrations were converted to melittin equivalents using the melittin content determined by UHPLC-ESI-MS (a factor of 0.6082). Points represent mean ± SD (*n* = 4).

**Figure 5:**
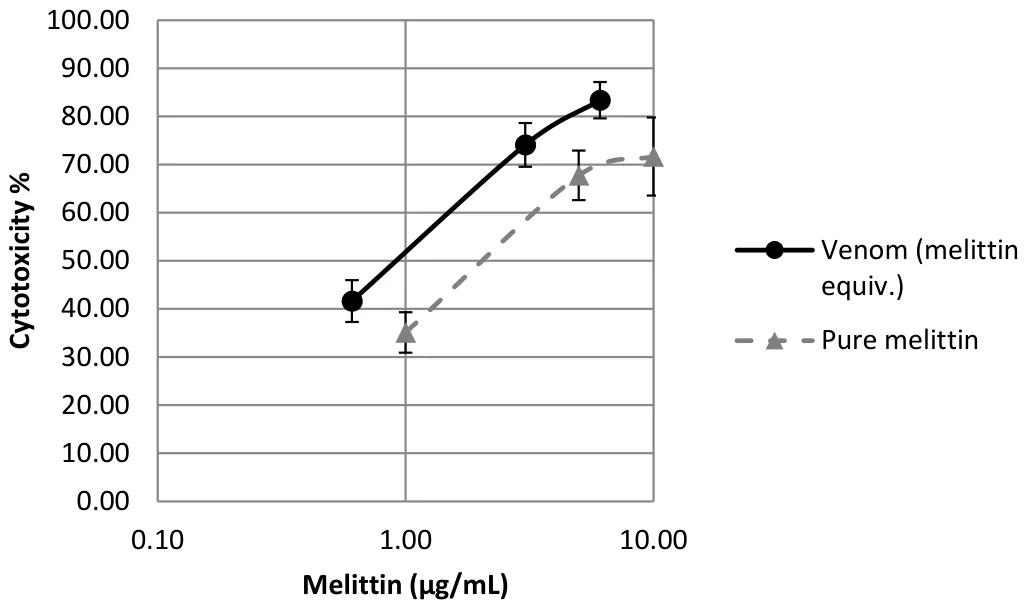
Dose-dependent effects of bee venom (expressed as melittin-equivalent concentration) and pure melittin on HEKa cell cytotoxicity determined by the LDH assay. Bee venom concentrations were converted to melittin equivalents using the melittin content determined by UHPLC-ESI-MS (a factor of 0.6082). Points represent mean ± SD (*n* = 4).

**Figure 6:**
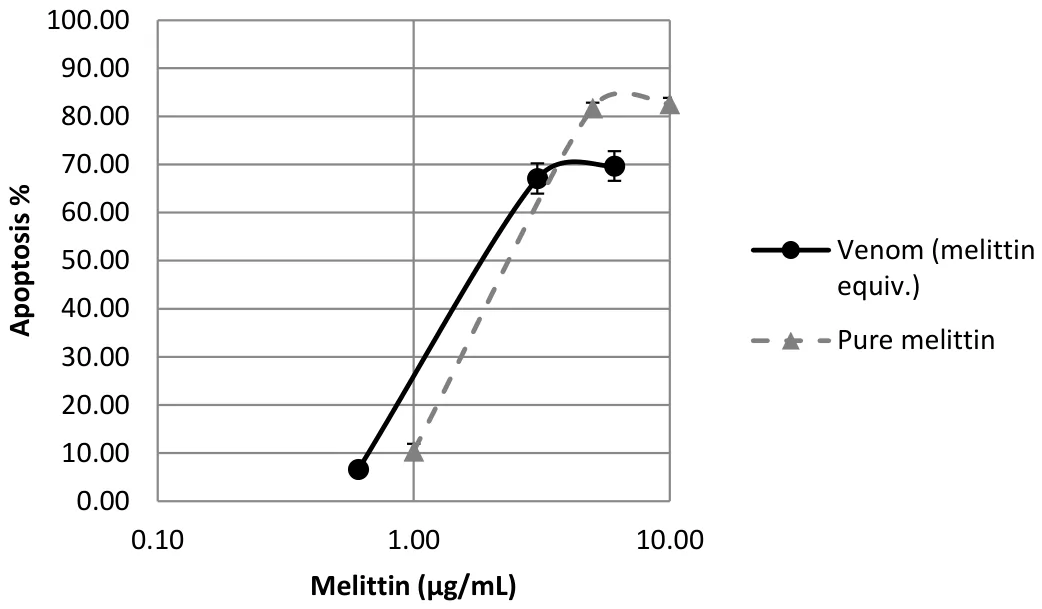
Dose-dependent effects of bee venom (expressed as melittin-equivalent concentration) and pure melittin on HEKa cell apoptosis determined by the FITC Annexin V Apoptosis Detection assay. Bee venom concentrations were converted to melittin equivalents using the melittin content determined by UHPLC-ESI-MS (a factor of 0.6082). Points represent mean ± SD (*n* = 3).

Two complementary dosing schemes were used. First, bee venom and the isolated peptides were compared at equal total concentrations (1, 5 and 10 µg/mL), which tests whether whole venom reproduces the activity of its dominant peptide at a matched total dose. Second, to account for the differing melittin content of these preparations, bee venom concentrations were recalculated to their melittin-equivalent content (×0.6082) and compared with pure melittin (Table 2 and Table S4). On this melittin-equivalent basis, bee venom reached the CC50 at approximately 1.3 µg/mL of melittin, whereas pure melittin required approximately 3.9 µg/mL (Figure 4), indicating that the cytotoxicity of whole venom exceeds that predicted from its melittin content alone and that additional venom constituents contribute to the effect. The relationship between melittin concentration and cytotoxicity assessed by the LDH assay is presented in Figure 5, whereas the correlation between melittin content and apoptosis induction is shown in Figure 6.

Comparison of the antibacterial activity reported above with the cytotoxicity data revealed that melittin inhibited bacterial growth and damaged keratinocytes within the same concentration range. Against the most susceptible strain (MRSE, MIC 2.5 µg/mL), the selectivity index (SI = CC50/MIC) reached only 1.6, and against both MSSA and MRSA (MIC 5 µg/mL), it fell to 0.8, indicating that keratinocyte damage occurred at or below the antibacterial concentrations. For standardized bee venom, the corresponding index against MRSE was 0.2. This narrow to absent therapeutic window does not support the direct topical application of melittin as an antibacterial agent and is consistent with the in vitro and in vivo toxicity reported for melittin against clinical extensively drug-resistant bacteria [16].

Two-way ANOVA (concentration × LPS) confirmed a strong concentration-dependent effect of both bee venom and melittin (η^2^ ≈ 0.98; *p* < 10^−16^), whereas only negligible effects were observed for apamin. The effect of LPS reached statistical significance for bee venom (MTT assay: *p* = 0.008 after Holm correction at 5 µg/mL, d = −3.7; significant concentration × LPS interaction), confirming attenuation of cytotoxicity at submaximal concentrations. In the LDH assay, a significant LPS effect was also observed for apamin (F = 13.5; *p* = 0.002), suggesting rather a general influence of LPS on the LDH signal than a compound-specific effect. The assumptions of normality and homogeneity of variance (Shapiro–Wilk and Levene tests) were fulfilled.

## 3. Discussion

The therapeutic application of bee venom requires standardization to ensure both its efficacy and safety. Due to the highly complex and variable composition of bee venom, which may differ depending on bee species, environmental conditions, collection methods, and storage parameters, the characterization of its major bioactive constituents is essential for the development of medicinal preparations. In particular, melittin is widely recognized as the principal biologically active peptide responsible for many of its biological effects. In contrast, other components, including apamin, may significantly influence both therapeutic activity and safety profiles. Therefore, quantitative and qualitative analysis of bee venom components using advanced analytical techniques is of particular importance. In the present study, the application of UHPLC–ESI–MS enabled precise determination of melittin and apamin content in the collected bee venom samples. Melittin was identified as the dominant component of bee venom, accounting for (60.82 ± 3.52)% *w*/*w*, whereas apamin was present at a substantially lower level of only (2.22 ± 0.17)% *w*/*w*; consequently, the melittin-to-apamin ratio was approximately 27:1. The quantified constituents accounted for approximately 63% of the total bee venom mass, while the remaining ~37% of the venom composition was not characterized in the present study. Quantification of melittin may be especially relevant considering its proposed role as a marker compound for bee venom standardization. The present study demonstrated that melittin was the principal antibacterial component of bee venom, whereas apamin showed no detectable antimicrobial activity at the tested concentrations. Moreover, bee venom exhibited selective activity only against methicillin-resistant *Staphylococcus epidermidis* MRSE. According to Acaroz et al., melittin inhibited *S. epidermidis* (MIC = 12.5–50 μg/mL) [17]. In our study, the MIC value determined for melittin was 2.5 µg/mL (Table 1), that is, approximately five- to twenty-fold lower, and therefore indicated somewhat higher potency. This difference may reflect the assay conditions, the peptide preparation, and the use of a clinical MRSE isolate rather than a reference strain. Importantly, despite the bactericidal profile of melittin (MBC/MIC ≤ 2), its antibacterial concentrations were markedly higher than those inducing cytotoxicity toward human keratinocytes, which substantially limits its direct topical therapeutic application. The obtained findings are consistent with previous reports demonstrating that melittin constitutes the major peptide of bee venom and is primarily responsible for its antibacterial activity. Leandro et al. [18] reported that melittin exhibited strong activity against oral Gram-positive pathogens, with MIC values ranging from 4 to 40 µg/mL, whereas phospholipase A2 showed minimal or no antibacterial activity (>400 µg/mL for most strains). Similarly, the review by El-Seedi et al. [12] summarized that melittin demonstrates broad-spectrum activity predominantly against Gram-positive bacteria, including methicillin-resistant *Staphylococcus aureus* (MRSA), *Enterococcus* spp., and oral streptococci. The increasing prevalence of multidrug-resistant (MDR) bacteria represents a major challenge for contemporary antimicrobial therapy. Melittin has been reported to exhibit broad antibacterial activity against methicillin-susceptible *Staphylococcus aureus* (MSSA), with MIC values ranging from 0.5 to 4 µg/mL [12]. The selective susceptibility of Gram-positive bacteria observed in the present study may be associated with structural differences in bacterial cell envelopes. Melittin is an amphipathic cationic peptide capable of inserting into lipid bilayers and disrupting membrane integrity through pore formation and membrane destabilization. The membrane-disruptive mechanism of melittin results in leakage of intracellular contents, cell lysis, and rapid bactericidal action, which is reflected by the low MBC/MIC ratio observed in the current study.

In contrast, apamin did not exhibit antibacterial activity under the tested experimental conditions. This observation is in agreement with the available literature, where apamin is mainly described as a neuroactive peptide acting on Ca^2+^-activated K^+^ channels rather than as an antimicrobial compound [19]. Although bee venom contains several biologically active peptides, not all contribute equally to antimicrobial effects, and the present findings further support the dominant antibacterial role of melittin.

Interestingly, bee venom in tested concentrations demonstrated antibacterial activity only against methicillin-resistant *Staphylococcus epidermidis*. This may reflect differences in bee venom composition, as it is a highly complex mixture containing peptides, enzymes, amines, and low-molecular-weight compounds, whose biological activity may vary depending on geographical origin, collection method, and processing conditions. Previous studies have shown that the antimicrobial activity of whole bee venom may differ considerably between bacterial strains and may be influenced by synergistic or antagonistic interactions among its constituents [12]. This is supported by another study, in which Tülin G. Gökmen et al. showed that BV collected from *Apis mellifera anatoliaca* in Turkey exhibited both a minimum inhibitory concentration and a minimum bactericidal concentration of 6.25 μg/mL against methicillin-resistant *Staphylococcus aureus* (MRSA) [20].

The *icaA* and *icaD* genes play a key role in staphylococcal biofilm formation [21]. They encode the enzymes responsible for the synthesis of polysaccharide intercellular adhesin (PIA), the primary component of the biofilm matrix [21].

The bacterial panel was deliberately restricted to staphylococci. The aim of the present study was not to characterize the broad-spectrum antimicrobial activity of bee venom and melittin, which has already been reported extensively against a wide range of microorganisms, but to relate antibacterial activity to keratinocyte safety within one skin-relevant model. Staphylococci naturally colonize human skin and form an important part of the normal skin microbiota, yet they also possess considerable pathogenic and opportunistic potential. Besides skin and soft tissue infections, they are important etiological agents of postoperative and surgical site infections, catheter-associated infections and infections related to implanted medical devices, including prosthetic joint infections. This dual role, as commensals and as clinically relevant opportunistic pathogens, provided the rationale for selecting a focused panel comprising MSSA, MRSA and MRSE.

Despite the promising antibacterial properties of melittin, the cytotoxicity results obtained in the present study constitute a major limitation. Melittin damaged keratinocytes at concentrations close to those required for bacterial growth inhibition. The resulting selectivity index (SI = CC50/MIC) reached 1.6 against the most susceptible strain, MRSE, and only 0.8 against both MSSA and MRSA, whereas standardized bee venom reached 0.2 against MRSE. Values at or near unity indicate that keratinocyte damage occurs at, or below, the antibacterial concentrations, which falls well short of the margin generally regarded as necessary for a therapeutic antimicrobial peptide. This narrow therapeutic window argues against the direct topical use of native melittin as an antibacterial agent. The high cytotoxicity of melittin toward mammalian cells is well documented and is closely related to its non-selective membranolytic mechanism. Melittin interacts not only with bacterial membranes but also with eukaryotic cell membranes, leading to membrane permeabilization, hemolysis, and cell death. Previous studies demonstrated that melittin exhibits substantial hemolytic activity, which further limits its clinical applicability [16]. Researchers highlight that melittin exerts potent pro-apoptotic activity through multiple mechanisms, including caspase activation, modulation of cell cycle progression, inhibition of proliferation, and induction of both apoptotic and necrotic cell death pathways. Moreover, melittin has been shown to influence matrix metalloproteinase activity, further contributing to its cytotoxic effects against cancer cells [9].

Melittin has been reported to act synergistically with conventional antibiotics against staphylococci, although no combination experiments were performed in the present study, and the following observations are therefore literature background rather than findings of this work. Synergy with oxacillin against MRSA, including inhibition of biofilm formation and a reduction in the concentrations required for an antibacterial effect, has been described [22], as has synergy with beta-lactams and aminoglycosides against MRSA [23]. Melittin also downregulated the biofilm-associated genes encoding intercellular adhesion A (*icaA*), accumulation-associated protein (*aap*), and phenol-soluble modulins (*psm*), all of which are involved in biofilm formation [24].

Whole bee venom behaved comparably, with a synergistic bactericidal effect against MRSA in combination with gentamicin or vancomycin [25]. Combinations of bee venom or of melittin with oxacillin were bactericidal against MRSA within 6 h and 4 h, respectively [26].

The present study demonstrates several limitations that should be considered when interpreting the obtained results. First, the antibacterial activity of standardized bee venom and its major components, melittin and apamin, was evaluated against a limited panel of staphylococcal strains, including methicillin-susceptible and methicillin-resistant *Staphylococcus aureus*, as well as methicillin-resistant *Staphylococcus epidermidis*. Therefore, the results cannot be directly extrapolated to other clinically relevant Gram-positive or Gram-negative pathogens. The panel was nevertheless selected deliberately to span a methicillin-susceptible and a methicillin-resistant phenotype relevant to skin and soft tissue infection, consistent with the intended topical context of the study, and its extension to a broader bacterial panel and to additional skin cell models, such as fibroblasts and reconstructed epidermis, is a defined direction for subsequent work. Second, keratinocyte responses were assessed in a single primary cell type. Third, although melittin demonstrated antibacterial activity, both bee venom and melittin exhibited pronounced cytotoxicity toward HEKa keratinocytes in MTT and LDH assays, accompanied by a high proportion of necrotic cells in Annexin V/PI analysis at concentrations of 5 and 10 µg/mL. These findings indicate a limited therapeutic applicability and suggest that the antimicrobial concentrations overlap with those inducing substantial damage to human keratinocytes. Finally, the in vitro experimental design does not fully reflect the complexity of the skin microenvironment, including immune responses, tissue penetration, and interactions with extracellular matrix components. Further studies employing checkerboard assays with determination of the fractional inhibitory concentration index, additional skin cell models, and in vivo models are required to better evaluate the therapeutic potential and safety profile of bee venom-derived peptides as antimicrobial agents.

Overall, the present study quantifies the therapeutic window of standardized bee venom and of its principal peptide within a single experimental framework. Melittin was the major antibacterial constituent of bee venom, but its pronounced cytotoxicity toward keratinocytes substantially limits its direct clinical applicability as a topical antibacterial agent. Future investigations should focus on improving the selectivity index of melittin-based therapies through structural modifications, formulation optimization, and combination approaches with conventional antimicrobials.

## 4. Materials and Methods

Bee venom was collected by Mirosław Szczepański in an apiary located in Garwolin, Poland. Melittin from honeybee venom and apamin were purchased from Merck KGaA (Darmstadt, Germany). Dulbecco’s Phosphate Buffered Saline (D-PBS) with calcium and magnesium was obtained from PAN Biotech (Aidenbach, Germany). Primary Epidermal Keratinocytes, Normal Human Adult (HEKa), PCS-200-011, Dermal Basal Medium (ATCC PCS-200-011), Keratinocyte Growth Kit (ATCC PCS-200-040), Penicillin–Streptomycin–Amphotericin B Solution (ATCC PCS-999-002), Trypsin-EDTA for Primary Cells (ATCC PCS-999-003), Trypsin Neutralizing Solution (ATCC PCS-999-004), and Dulbecco’s Phosphate Buffered Saline (D-PBS) without calcium and magnesium were provided from the American Type Culture Collection (ATCC, Manassas, VA, USA). Lipopolysaccharide (LPS; *E. coli* O26:B6) was obtained from Difco Laboratories (Detroit, MI, USA). A 0.2% solution of chlorhexidine digluconate was obtained from Chemidental (Pabianice, Poland). Propidium iodide (PI) and Annexin V-FITC were used (BD Pharmingen FITC Annexin V Apoptosis Detection Kit I, Becton Dickinson Biosciences, San Jose, CA, USA). The following bacterial strains were used in the microbiological assays: *Staphylococcus aureus* MSSA ATCC 29213—a reference strain obtained from the ATCC collection (American Type Culture Collection, Manassas, VA, USA); *Staphylococcus aureus* MRSA—a clinical isolate recovered from a patient with a surgical site infection; and *Staphylococcus epidermidis* MRSE—a clinical isolate obtained from a patient with a skin and soft tissue infection. The clinical isolates were identified, and their species identity was confirmed using matrix-assisted laser desorption/ionization time-of-flight mass spectrometry (MALDI-TOF MS; VITEK MS PRIME, bioMérieux, Marcy-l’Étoile, France). Drug sensitivity of the tested staphylococcal strains was determined according to the EUCAST guidelines version 16.1 [27] by an automatic method using VITEK 2 COMPACT (bioMérieux, Marcy-l’Étoile, France), and a disk diffusion test with cefoxitin was used to detect methicillin resistance (Supplementary Materials Table S6).

### 4.1. Collection of Bee Venom

The bee venom (BV) was collected from an apiary with the following GPS geolocation coordinates: 51.848733, 21.466052. For scientific purposes, BV was collected with a special device made of glass panels and wires emitting a low-voltage current from 18 to 22 V. This device was placed into the beehive. Under the influence of an electric field, irritated bees secrete their venom directly onto the slide, from which venom is scraped after drying. The bee venom powder was kept at −80 °C for further use. Bee venom was dissolved in D-PBS to obtain a stock solution of 1 mg/mL and filtered through a 0.22 µm filter. The solution was kept at −20 °C for further use.

### 4.2. Preparation of Bee Venom Samples

The quantification of melittin and apamin in the venom was conducted after mixing 50 µL of stock solution with 950 µL of deionized water. For the quantification of apamin, the injection of 6 µL of the sample was performed, while for the quantification of melittin, 1 µL was used. The analysis was performed in triplicate.

### 4.3. UHPLC-ESI-MS Analysis

The analysis was conducted via UHPLC utilizing the Ultimate 3000 series system (Dionex, Idstein, Germany), equipped with a dual low-pressure gradient pump with vacuum degasser, an autosampler, and a column compartment coupled with an Amazon SL ion trap mass spectrometer (Bruker Daltonik GmbH, Bremen, Germany). Compound separation within the analyzed extracts was obtained on a Hypersil Gold C18 column (50 mm × 2.1 mm × 1.9 μm; Thermo Fisher Scientific, Waltham, MA, USA), with the column temperature maintained at 25 °C. Elution was performed using mobile phase A (0.1% formic acid in deionized water) and mobile phase B (0.1% formic acid in acetonitrile) via a multi-step gradient: starting at 5% B for 0 min and increasing to 70% B in 15 min. The flow rate was set to 0.300 mL/min. Different volumes of venom sample or calibration solution were injected by the autosampler onto the column. Equilibration of the column was maintained for 7 min between injections. The eluate was directly introduced into the mass spectrometer without splitting. The ion trap Amazon SL mass spectrometer was equipped with an ESI interface, with the following parameters: nebulizer pressure of 40 psi, dry gas flow of 9 L/min, dry temperature of 135 °C, and capillary voltage of 4.5 kV. Analysis was conducted via scan from *m*/*z* 70–2200, with compounds analyzed in positive ion mode. The instrument was used in the smart parameter setting mode for *m*/*z* = 500.

### 4.4. Preparation of Chemical Standards

Melittin (≥85%, HPLC grade, Cat. No. M2272) and apamin (≥98%, HPLC, Cat. No. 178270), both from Merck KGaA (Darmstadt, Germany), were dissolved in D-PBS to obtain a stock solution of 1 mg/mL. The solution was kept at −20 °C for further use. To establish calibration curves, the stock solution was diluted with deionized water. A 50 µL measure of an apamin stock solution and a 50 µL measure of a melittin stock solution were added to 900 µL of deionized water to obtain a solution at a concentration of 50 µg/mL of each compound. The solution was used for calibration using UHPLC-ESI-MS analysis.

### 4.5. Calibration Curves

To plot calibration curves for the quantification of both compounds, injections of different volumes of stock solution were used (0.1, 0.25, 0.5, 1.0, 2.0, 4.0 and 6.0 µL). The calibration curves were prepared using extracted ion chromatograms (EICs) for apamin and melittin. Most characteristic ions in the MS spectra of both compounds were chosen based on a previous report [28]. These were *m*/*z* = 406.3, 507.7 and 676.7 for apamin and *m*/*z* = 475.3, 570.2, 712.5 and 949.8 for melittin. Calibration curves were plotted over the range of 5 to 300 ng per injection for both compounds. Next, the two upper calibration points were dismissed in order to obtain linear fitting in both cases. The final linearity range was 5–100 ng per injection with the following equations: apamin—y = 12,349,692.2x + 33,554,089.2 and melittin—y = 56,660,221.8x + 80,515,797.7. The R-values were 0.9998 and 0.9994, respectively.

### 4.6. The Cultivation of HEKa

Primary Epidermal Keratinocytes, Normal Human Adult (HEKa), were cultured under sterile conditions in 25 cm^2^ flasks and were subsequently transferred into 75 cm^2^ culture flasks. The commercial growth medium, Dermal Basal Medium with Keratinocyte Growth Kit, which contains Bovine Pituitary Extract, rh TGF-α, L-glutamine, hydrocortisone hemisuccinate, insulin, epinephrine and apo-transferrin, was used. In order to ensure proper growth, Penicillin-Streptomycin-Amphotericin B Solution was added to the growth medium. Cells were cultured at 37 °C. The monolayer was rinsed with D-PBS without calcium and magnesium ions to remove residual culture medium. The cells were detached with trypsin (0.05%)-EDTA (0.02%) for 3 min. Subsequently, an equal volume of trypsin neutralizing solution was added. After centrifugation at 125× *g* MPW-351RH (MPW Med. Instruments, Warsaw, Poland), a cell suspension with a density of 100,000 cells/mL was prepared and seeded into a 96-well plate, resulting in a density of 20,000 cells per well. The cell counting was performed using a Bürker chamber according to the methodology described by Kurek-Górecka et al. [29]. The prepared 96-well plates were incubated at 37 °C in an atmosphere containing 5% CO_2_ for 24 h. After 24 h, the cells were stimulated with the tested BV, melittin and apamin at the following concentrations: 10 µg/mL, 5 µg/mL, and 1 µg/mL. Previously, the dried bee venom was dissolved in D-PBS to prepare various final concentrations: 10 µg/mL, 5 µg/mL and 1 µg/mL.

### 4.7. Cell Viability (MTT) Assay

A colorimetric MTT assay was used to assess the viability of cells after stimulation to the BV, melittin, and apamin solution in D-PBS. The HEKa cells were treated with a range of concentrations of the analyzed solutions (10, 5 and 1 μg/mL) and the control (medium). Bee venom and the isolated peptides were applied at equal total concentrations (10, 5 and 1 µg/mL) so that whole venom could be compared with its dominant peptide at a matched total dose. Because the analyzed bee venom contained 60.82% melittin by mass, the bee venom concentrations were additionally recalculated to their melittin-equivalent content (a factor of 0.6082) and compared with the corresponding concentrations of pure melittin. Both dosing schemes are reported for the MTT, LDH and apoptosis assays. Cell viability was expressed as a percentage of the vehicle control assayed on the same plate, with the highest vehicle-control well of each plate taken as 100%. Bee venom and apamin were assayed on one plate and melittin on a separate plate, and each compound was referenced to the vehicle control of its own plate. After 24 h of treatment, 20 μL of MTT solution (5 mg/mL) was added to each well and incubated for 4 h at 37 °C. MTT is reduced to purple formazan crystals by metabolically active cells. The insoluble formazan crystals were dissolved in DMSO, and the absorbance was measured at 520 nm. Each treatment was tested in four replicate wells.

### 4.8. The Cytotoxicity Assay—LDH Assay

In this assay, lactate dehydrogenase (LDH) activity was determined using an enzymatic test in which NAD+ was reduced to NADH/H+ during the LDH-catalyzed conversion of lactate to pyruvate. In the second step of the reaction, two hydrogen atoms are transferred from NADH/H+ to the yellow tetrazolium salt INT (2-[4iodophe-nyl]-3-[4-nitrophenyl]-5-phenyltetrazolium chloride) by a catalyst. Cytotoxicity was assessed by measuring LDH release into the culture supernatant as an indicator of plasma membrane damage. An increase in LDH activity reflected an elevated number of dead or membrane-compromised cells. Cytotoxicity was expressed relative to the maximum LDH release obtained with 2% Triton X-100, after subtraction of the background absorbance of cell-free medium. The spontaneous LDH release of the vehicle control was not subtracted and is therefore included in the reported values, the vehicle control corresponding to (32.4 ± 4.5)% on this scale. The enzymatic activity in the supernatant was directly proportional to the amount of formazan generated during a defined incubation period. Thus, the intensity of color developed in the assay corresponded to the number of lysed cells. The water-soluble formazan product was quantified spectrophotometrically at approximately 490 nm, a wavelength at which the tetrazolium salt exhibits no significant absorbance. BV, MEL and APA at the concentrations of 10, 5, and 1 μg/mL, with or without LPS, were added to 96 wells. A 2% Triton X-100 solution was added to the wells with cell culture. Then, 20 μL of supernatant, 80 μL of distilled water and 100 μL of reaction mixture were combined in wells. Two controls were included in the experimental model: the high control (lysed cells) and the low control (medium). After 10 min of incubation, to each well, 0.5M H_2_SO_4_ was added to each well to stop the reaction. Absorbance was then measured at 490 nm.

Percentage cytotoxicity was calculated from the measured absorbance values according to Formula (1).

Cytotoxicity [%] = (experimental value − low control)/(high control − low control) × 100(1)

### 4.9. FITC Annexin V Apoptosis Detection Assay

To determine whether BV and its melittin and apamin components induced apoptosis and to distinguish necrotic cells from apoptotic cells, a fluorescent staining assay using propidium iodide (PI) and Annexin V-FITC was conducted (BD Pharmingen FITC Annexin V Apoptosis Detection Kit I, Becton Dickinson Biosciences, San Jose, CA, USA). HEKa cells were seeded in a cell culture plate, 24-well (Nunc™, Thermo Fisher Scientific, Waltham, MA, USA), at a density of 6 × 10^4^ cells/mL and allowed to adhere for 72 h. The cells were then treated for 24 h with 10, 5, and 1 μg/mL of BV, melittin, and apamin and with 250 and 100 μM camptothecin (CPT, as positive control) or medium (negative control).

PI is a membrane-impermeable fluorescent dye that binds to DNA and stains cells with disrupted membrane integrity, including late apoptotic and necrotic cells. In contrast, Annexin V is used to detect apoptotic cells by its ability to bind to phosphatidylserine (PS), an early marker of apoptosis that changes its location in the cell membrane, moving from the inner to the outer layer. To enable detection in a flow cytometer, Annexin V conjugated to FITC (fluorescein isothiocyanate, a green fluorescent dye) was used.

After this time, cells were harvested using trypsin and ethylenediaminetetracetic acid (EDTA) (Trypsin-EDTA for primary cells, ATCC), and after 10 min, a trypsin neutralizing solution was added (Trypsin neutralizing solution, ATCC). Cells were then washed twice with Dulbecco’s phosphate-buffered saline (D-PBS) solution and resuspended in 1× Annexin V Binding Buffer (100 µL). Then the cell suspension was incubated with Annexin V-FITC (5 µL) and PI (5 µL) for 15 min at RT in the dark.

After this time, 400 μL of 1× Binding Buffer was added to each tube. The cells were analyzed by flow cytometry (LSR II, Becton Dickinson Biosciences, San Jose, CA, USA) within 1 h. During the flow cytometry analysis, 10,000 events were collected from each sample tube.

### 4.10. Determination of the Minimum Inhibitory Concentration (MIC) and Minimum Bactericidal Concentration (MBC) of BV, Melittin and Apamin

In the conducted study, the antimicrobial activity of the bee venom, melittin and apamin was evaluated using the minimum inhibitory concentration (MIC) assay. The MIC is the lowest concentration of antimicrobial agent that completely inhibits the growth of organisms in microdilution wells or in tubes after 16 ± 2 h of incubation at 35 °C. MIC values were determined by broth microdilution in 96-well microtiter plates. The bee venom was dissolved in D-PBS with calcium and magnesium. Serial dilutions of the BV, melittin and apamin were then performed to obtain different final concentrations: 10, 5, 2.5, 1.25, 0.62, 0.31, 0.155, 0.078, 0.039, and 0.02 µg/mL. For each bacterial strain (*Staphylococcus aureus* MSSA, *Staphylococcus aureus* MRSA, and *Staphylococcus epidermidis* MRSE), a bacterial inoculum of 0.5 McFarland turbidity was prepared by suspending colonies in 0.9% NaCl solution. Brain Heart Infusion (BHI) broth was used as the growth medium for all bacterial species. The examined wells contained 160 µL of appropriate broth, 20 µL of the analyzed substances, and 20 µL of a bacterial suspension at the final density of 5 × 10^5^ CFU/mL. Positive and negative controls were included on each plate. A 0.2% solution of chlorhexidine digluconate (Chemidental, Pabianice, Poland) with tested strains was used as a positive control. The negative control consisted of suspensions of the tested staphylococcal strains in Brain Heart Infusion broth. For the minimum bactericidal concentration (MBC) assessment, 20 µL of liquid culture that showed no visible growth in the MIC assay was taken and inoculated onto fresh blood agar plates. The plates were then incubated at 37 °C for 24 h. The minimum bactericidal concentration was determined as the lowest concentration showing no visible growth on plates.

### 4.11. Statistical Analysis

Statistical analyses were performed using Statistica 13.3 software and Microsoft Excel. Data were expressed as mean ± standard deviation (SD); the sample size was *n* = 4 for the MTT and LDH assays and *n* = 3 for the flow cytometric analysis (Annexin V/PI). Normality of data distribution was verified using the Shapiro–Wilk test, while homogeneity of variances was assessed with Levene’s test; the significance level was set at α = 0.05. The effects of concentration and co-stimulation with lipopolysaccharide (LPS) were evaluated by two-way analysis of variance (Type II ANOVA, concentration × LPS design), performed separately for each tested substance, with effect size reported as η^2^. Multiple comparisons versus the control group (DMSO control) were conducted using Dunnett’s test, whereas differences between the −LPS and +LPS conditions were analyzed using Welch’s *t*-test with Holm correction, reporting Cohen’s d effect size. Agreement between assays was assessed using Spearman’s rank correlation coefficient (ρ). CC50 values were determined by log-linear interpolation between concentrations surrounding 50% viability. Exact two-sided *p*-values were reported, with values below 0.001 presented as *p* < 0.001. Differences were considered statistically significant at *p* < 0.05.

## 5. Conclusions

This study standardized bee venom to its melittin and apamin content by UHPLC-ESI-MS and used that standardization to separate two questions that are often conflated, namely how reproducible and potent a venom batch is, and how safe it is. Melittin was confirmed as the principal antibacterial constituent, active against the tested staphylococci, whereas apamin showed no antibacterial activity in the concentration range studied. Cytotoxicity toward human keratinocytes, however, did not follow melittin content alone. On a melittin-equivalent basis, whole venom was more cytotoxic than pure melittin, which indicates that constituents other than melittin contribute to the cytotoxic effect.

This observation is the central message of the study. Quantifying melittin is necessary to ensure batch-to-batch reproducibility and to predict the potency of a bee venom preparation, but it is not sufficient to predict its safety, because the cytotoxic burden of the whole venom exceeds what its melittin content alone would suggest. For the increasing use of bee venom in dermatological and cosmetic formulations, this means that safety must be assessed on the standardized whole venom rather than inferred from a single marker peptide.

Melittin itself, despite its antibacterial activity, damaged keratinocytes at concentrations close to or below those required for bacterial inhibition, so its direct topical use as an antibacterial agent is not supported. Validated analytical standardization such as UHPLC-ESI-MS profiling, combined with safety evaluation of the whole venom, therefore represents a rational basis for developing reproducible, medical-grade bee venom preparations. These conclusions apply to the three staphylococcal strains and to the primary keratinocyte model examined here.

## Figures and Tables

**Table 1:** Minimum inhibitory (MIC) and minimum bactericidal (MBC) concentrations of standardized bee venom (BV), melittin and apamin against the tested staphylococcal strains, determined by broth microdilution over the range of 0.02 to 10 µg/mL. Chlorhexidine digluconate (0.2% stock solution) served as the positive control and BHI broth as the negative control. Values given as >10.00 indicate that no inhibition or no bactericidal effect was observed at the highest concentration tested. An MBC/MIC ratio of 2 or below was taken to indicate a bactericidal effect.

Analyzed Substance	*Staphylococcus aureus* MSSA		*Staphylococcus aureus* MRSA		*Staphylococcus epidermidis* MRSE	
	MIC [µg/mL]	MBC [µg/mL]	MIC [µg/mL]	MBC [µg/mL]	MIC [µg/mL]	MBC [µg/mL]
Bee venom	>10.00	>10.00	>10.00	>10.00	10.00	10.00
Melittin	5.00	5.00	5.00	5.00	2.50	5.00
Apamin	>10.00	>10.00	>10.00	>10.00	>10.00	>10.00
Chlorhexidine	1.55	3.10	1.55	6.25	1.55	3.1

**Table 2:** Half-maximal cytotoxic concentrations (CC50) of bee venom, melittin and apamin in HEKa keratinocytes, determined from the MTT assay by log-linear interpolation between the concentrations bracketing 50% viability. Values are given both as the total concentration of the tested preparation and as the melittin-equivalent concentration, obtained by multiplying the bee venom concentration by its melittin content (a factor of 0.6082). “Not applicable” indicates that the CC50 was not reached within the tested concentration range, that is, up to 10 µg/mL. The same data are provided in Supplementary Table S4.

Compound	CC50, −LPS (µg/mL)	CC50, +LPS (µg/mL)	CC50 as Melittin Equivalent (µg/mL)	Note
Bee venom	2.18	2.95	1.33	Venom contains 60.8% melittin (factor 0.6082)
Melittin	3.91	4.48	3.91	Pure compound
Apamin	>10	>10	Not applicable	CC50 not reached

## Data Availability

The raw per-well data supporting the reported results are provided in the Supplementary Materials (Table S13). Further data are available from the corresponding author on reasonable request.

## Supplementary Materials

The following supporting information can be downloaded at https://www.mdpi.com/article/10.3390/molecules31162787/s1.

## Abbreviations

The following abbreviations are used in this manuscript:

BV	Bee venom
MEL	Melittin
APA	Apamin
PLA2	Phospholipase A2
MCDP	Mast cell degranulating peptide
MRJP	Major royal jelly proteins
Api m 5	Dipeptidyl peptidase IV
Api m 6	Serine protease inhibitor
Api m 7	CUB serine protease
Api m 10	Icarapin
EUCAST	European Committee on Antimicrobial Susceptibility Testing
IL-6	Interleukin 6
IL-8	Interleukin 8
PDGF	Platelet-Derived Growth Factor
NF-ĸB	Nuclear factor kappa-light-chain-enhancer of activated B cells
TLR	Toll-like receptor
MAPK	Mitogen-activated protein kinase
CV	coefficient of variation
MTT	3-(4,5-dimethylthiazol-2-yl)-2,5-diphenyltetrazolium bromide
LDH	Lactate dehydrogenase
LPS	Lipopolysaccharide
INT	2-[4iodophenyl]-3-[4-nitrophenyl]-5-phenyltetrazolium chloride
HEKa	Primary Epidermal Keratinocytes Normal Human Adult
UHPLC-ESI-MS	Ultra-High Performance Liquid Chromatography coupled with Electrospray Ionization Mass Spectrometry
MIC	Minimum inhibitory concentration
MBC	Minimum bactericidal concentration
MSSA	Methicillin-susceptible *Staphylococcus aureus*
MRSA	Methicillin-resistant *Staphylococcus aureus*
MRSE	Methicillin-resistant *Staphylococcus epidermidis*
CHX	Chlorhexidine
BHI	Brain heart infusion
*icaA*	intercellular adhesion A
*aap*	accumulation-associated protein
*psm*	phenol-soluble modulins
