# Supplementary material for: Melittin- and Apamin-Standardized Bee Venom: UHPLC-ESI-MS Characterization, Antibacterial Activity, and Apoptosis Induction in Human Keratinocytes
Source: Molecules. 2026 Aug 11;31(16):2787. doi: 10.3390/molecules31162787 (PMC13515527; doi:10.3390/molecules31162787)
Supplement: Supplementary file 1 [file molecules-31-02787-s001.zip › molecules-4435045-supplementary.pdf]

Supplementary Materials

Melittin- and Apamin-Standardized Bee Venom: UHPLC-ESI-MS Characterization, Antibacterial Activity, and Apoptosis Induction in Human Keratinocytes

Anna Kurek-Górecka [1,*], Małgorzata Kłósek [1], Dagmara Jaworska [1], Anna Mertas [1], Radosław Balwierz [2], Sebastian Granica [3], Mirosław Szczepański [4], Katarzyna Piszczatowska [4], Sevgi Kolayli [5,6] and Zenon P. Czuba [1]

1 Department of Microbiology and Immunology, Faculty of Medical Sciences in Zabrze, Medical University of Silesia in Katowice, Jordana 19, 41-808 Zabrze, Poland; mklosek@sum.edu.pl (M.K.); djaworska@sum.edu.pl (D.J.); amertas@sum.edu.pl (A.M.); zczuba@sum.edu.pl (Z.P.C.)

2 Institute of Chemistry, University of Opole, Oleska 48, 45-052 Opole, Poland; radoslaw.balwierz@uni.opole.pl

3 Department of Pharmaceutical Biology, Medical University of Warsaw, 1 Banacha St., 02-097 Warsaw, Poland

4 Department of Biochemistry, Medical University of Warsaw, 02-091 Warsaw, Poland; mszczepanski@wum.edu.pl (M.S.); katarzyna.piszczatowska@wum.edu.pl (K.P.)

5 Department of Chemistry, Faculty of Science, Karadeniz Technical University, 61080 Trabzon, Türkiye; skolayli@ktu.edu.tr

6 Faculty of Medicine, Nakhchivan State University, Nakhchivan AZ7012, Azerbaijan

* Correspondence: akurekgorecka@sum.edu.pl

Contents

Table S1. UHPLC–ESI–MS quantification and method validation (melittin, apamin).
Table S2. Cell viability (MTT) of HEKa keratinocytes, ±LPS.
Table S3. Cytotoxicity (LDH release) of HEKa keratinocytes, ±LPS.
Table S4. Half-maximal cytotoxic concentrations (CC50) and melittin equivalents.
Table S5. Apoptosis/necrosis quadrant distribution (Annexin V-FITC/PI).
Table S6. Drug sensitivity of the tested staphylococcal strains (S – susceptible; I – susceptible, increased exposure; R – resistant).
Table S7. Minimum inhibitory and bactericidal concentrations (MIC, MBC).
Table S8. Selectivity index (antibacterial vs cytotoxic activity).
Table S9. Two-way ANOVA (concentration × LPS).
Table S10. Pairwise −LPS vs +LPS comparisons (Welch t-test, Holm).
Table S11. Comparison to control (one-way ANOVA + Dunnett).
Table S12. Inter-assay correlation analysis (Spearman).
Table S13. Raw per-well data (MTT and LDH).
Figure S1. Calibration curve – melittin.
Figure S2. Calibration curve – apamin.
Figure S3. ESI-MS+ spectrum of melittin (m/z 475.38, 570.44, 712.61).
Figure S4. ESI-MS+ spectrum of apamin (m/z 507.70).
Figure S5. UHPLC–ESI–MS chromatograms of the apamin and melittin standards (BPC and EIC).
Figure S6. UHPLC–ESI–MS chromatograms of the bee venom sample (BPC and EIC).
Figure S7. HEKa cells following stimulation with bee venom (BV) at different concentrations: (A) 1 µg/mL, (B) 5 µg/mL, and (C) 10 µg/mL.
Figure S8. HEKa cells following stimulation with melittin (MEL) at different concentrations: (A) 1 µg/mL, (B) 5 µg/mL, and (C) 10 µg/mL.
Figure S9. HEKa cells following stimulation with apamin (APA) at different concentrations: (A) 1 µg/mL, (B) 5 µg/mL, and (C) 10 µg/mL.
Figure S10. HEKa cells following stimulation with camptothecin.
Figure S11. Apoptotic effect (showed as dot plots) of BV, melittin and apamin (1, 5, 10 µg/mL) in HEKa cells. Camptothecin (100, 250 µM) was used as positive control. Q1 – dead cells, Q2 – late apoptosis, Q3 – early apoptosis, Q4 – non-apoptotic cells.
Figure S12. Representative MIC microtiter plates (MRSA, MSSA, MRSE).
Figure S13. Representative MBC blood-agar plates – bee venom and melittin.
Figure S14. Representative MBC blood-agar plates – chlorhexidine.

1. Analytical characterization (UHPLC–ESI–MS)

Table S1. UHPLC–ESI–MS quantification and method validation for melittin and apamin in the standardized bee venom (n = 6 injections). Calibration range 5–300 ng per injection; the two uppermost points were excluded to obtain linearity over 5–100 ng. Calibration equations: melittin, y = 56660221.8x + 80515797.7; apamin, y = 12349692.2x + 33554089.2. The quantified peptides accounted for ≈ 63% of the total venom mass; the remaining ≈ 37% was not characterized.

Analyte	Content (µg/mg) mean ± SD	Content (% w/w) mean ± SD	CV (%)	Linear range (ng)	R	Characteristic ions (m/z)
Melittin	608.21 ± 35.16	60.82 ± 3.52	5.78	5–100	0.9994	475.3, 570.2, 712.5, 949.8
Apamin	22.17 ± 1.73	2.22 ± 0.17	7.82	5–100	0.9998	406.3, 507.7, 676.7
**Melittin : apamin ratio (w/w)**	**≈ 27 : 1**					

2. Cytotoxicity and viability

Table S2. Viability of HEKa keratinocytes after 24 h treatment, without (−LPS) and with (+LPS) lipopolysaccharide co-stimulation, by the MTT assay, expressed as percentage of the untreated control (n = 4). Control denotes the vehicle (DMSO) control (−LPS column) and the LPS-only control (+LPS column). Per ISO 10993-5:2009, viability above 70% is non-cytotoxic.

Compound	Concentration (µg/mL)	Viability % (−LPS) mean ± SD	Viability % (+LPS) mean ± SD
Bee venom	Control	98.55 ± 1.53	97.66 ± 1.75
	1	71.10 ± 0.87	72.14 ± 1.56
	5	27.51 ± 3.68	39.25 ± 2.54
	10	12.32 ± 0.98	12.13 ± 0.95
Melittin	Control	90.14 ± 7.13	85.44 ± 14.34
	1	86.87 ± 3.48	90.54 ± 2.11
	5	43.38 ± 2.75	47.04 ± 2.75
	10	29.52 ± 5.56	29.20 ± 4.03
Apamin	Control	98.55 ± 1.53	97.66 ± 1.75
	1	95.07 ± 1.56	94.14 ± 5.29
	5	87.35 ± 5.02	88.57 ± 3.81
	10	90.45 ± 4.67	89.38 ± 3.39

Table S3. Lactate dehydrogenase (LDH) release from HEKa keratinocytes after 24 h treatment, without (−LPS) and with (+LPS) LPS co-stimulation, expressed as percentage cytotoxicity relative to the high (lysed-cell) and low (medium) controls (n = 4). Control denotes the vehicle (DMSO) control.

Compound	Concentration (µg/mL)	Cytotoxicity % (−LPS) mean ± SD	Cytotoxicity % (+LPS) mean ± SD
Bee venom	Control	32.42 ± 4.53	30.83 ± 0.37
	1	41.63 ± 4.35	39.74 ± 3.24
	5	74.11 ± 4.55	60.51 ± 6.53
	10	83.40 ± 3.79	84.70 ± 7.20
Melittin	Control	46.74 ± 5.10	36.34 ± 4.16
	1	35.11 ± 4.20	32.82 ± 3.53
	5	67.77 ± 5.15	69.96 ± 7.97

Compound	Concentration (µg/mL)	Cytotoxicity % (−LPS) mean ± SD	Cytotoxicity % (+LPS) mean ± SD
	10	71.69 ± 8.13	72.93 ± 12.83
Apamin	Control	32.42 ± 4.53	30.83 ± 0.37
	1	40.65 ± 5.38	32.66 ± 4.04
	5	39.98 ± 3.45	36.02 ± 3.46
	10	44.83 ± 4.46	39.18 ± 1.68

Table S4. CC50 values (MTT assay) by log-linear interpolation between the concentrations bracketing 50% viability. The melittin-equivalent CC50 of bee venom (1.33 µg/mL) is lower than that of pure melittin (3.91 µg/mL), indicating that the cytotoxicity of bee venom is at least comparable to — and potentially greater than — that predicted from its melittin content alone.

Compound	CC50 −LPS (µg/mL)	CC50 +LPS (µg/mL)	CC50 in melittin-equivalent (µg/mL)	Note
Bee venom	2.18	2.95	1.33	venom ≡ 60.8% melittin (×0.6082)
Melittin	3.91	4.48	3.91	pure compound
Apamin	> 10	> 10	—	CC50 not reached

3. Apoptosis and necrosis (Annexin V-FITC / PI)

Table S5. Quadrant distribution of HEKa keratinocytes after 24 h treatment, by Annexin V-FITC/PI flow cytometry (n = 3). Early apoptotic = Annexin V^+/PI^- (Q4); late apoptotic = Annexin V^+/PI^+ (Q2); necrotic = Annexin V^-/PI^+ (Q1). Total apoptosis = early + late. The predominance of the late-apoptotic/necrotic fractions for bee venom and melittin is consistent with a membranolytic (pore-forming) mode of cell death.

Compound	Conc. (µg/mL)	Early apoptotic Q4 % (mean±SD)	Late apoptotic Q2 % (mean±SD)	Total apoptotic Q2+Q4 % (mean±SD)	Necrotic Q1 % (mean±SD)
Bee venom	1	0.70 ± 0.00	5.93 ± 0.75	6.63 ± 0.75	1.83 ± 0.35
	5	0.27 ± 0.12	66.83 ± 3.02	67.10 ± 3.13	22.70 ± 4.05
	10	0.23 ± 0.21	69.47 ± 3.27	69.70 ± 3.08	26.63 ± 1.20
Melittin	1	0.87 ± 0.29	9.57 ± 1.31	10.43 ± 1.52	1.00 ± 0.17
	5	3.67 ± 0.42	78.10 ± 1.10	81.77 ± 1.08	9.40 ± 1.83
	10	1.30 ± 0.10	81.33 ± 1.30	82.63 ± 1.25	12.93 ± 0.90
Apamin	1	2.50 ± 0.00	10.10 ± 0.30	12.60 ± 0.30	1.07 ± 0.15
	5	2.50 ± 0.10	10.37 ± 1.00	12.87 ± 0.90	0.77 ± 0.06
	10	2.27 ± 0.06	13.33 ± 1.06	15.60 ± 1.01	1.30 ± 0.10

4. Antibacterial activity

Table S6. Drug sensitivity of the tested staphylococcal strains (S – susceptible; I – susceptible, increased exposure; R – resistant).

ANTIBIOTIC	*S. aureus* MSSA ATCC 29213	*S. aureus* MRSA	*S. epidermidis* MRSE
Oxacillin	S	R	R
Cefoxitin	S	R	R
Ciprofloxacin	I	R	R
Levofloxacin	I	R	R
Moxifloxacin	S	R	R
Norfloxacin	S	R	R
Amikacin	S	S	R
Gentamicin	S	S	R
Tobramycin	S	R	R
Teicoplanin	S	S	S
Vancomycin	S	S	S
Erythromycin	S	R	S
Clindamycin	S	R	R
Tetracycline	S	S	S
Tigecycline	S	S	S
Linezolid	S	S	R
Daptomycin	S	S	S
Fusidic acid	S	S	S
Rifampicin	S	S	S
Trimethoprim	S	S	R
Trimethoprim-sulfamethoxazole	S	S	R

Table S7. Minimum inhibitory concentration (MIC) and minimum bactericidal concentration (MBC) by broth microdilution (BHI broth, 5×10^5 CFU/mL). Chlorhexidine digluconate served as the positive control. Melittin was the only active venom constituent (MBC/MIC ≤ 2, bactericidal); bee venom was active only against *S. epidermidis* MRSE; apamin was inactive. *Units are µg/mL, consistent with the serial-dilution range (10-0.02 µg/mL) given in Materials and Methods and with the selectivity calculation in Table S8.*

Compound	*S. aureus* MSSA		*S. aureus* MRSA		*S. epidermidis* MRSE	
	**MIC**	**MBC**	**MIC**	**MBC**	**MIC**	**MBC**
Bee venom	> 10	> 10	> 10	> 10	10	10
Melittin	5	5	5	5	2.5	5
Apamin	> 10	> 10	> 10	> 10	> 10	> 10
Chlorhexidine (0.2%)	1.55	3.1	1.55	6.25	1.55	3.1

Table S8. Selectivity assessment for the most susceptible strain (*S. epidermidis* MRSE). MIC values (Table S7) and CC50 values (Table S4) are both expressed in µg/mL and are therefore directly comparable. The selectivity index (SI = CC50/MIC) is 1.6 for melittin and 0.2 for bee venom. Values at or near unity indicate that keratinocyte damage occurs at, or below, the antibacterial concentrations, which corresponds to a narrow to absent therapeutic window. For melittin, the corresponding index against S. aureus MSSA and MRSA (MIC 5 µg/mL) is 0.8.

Compound	MIC vs MRSE (µg/mL)	CC50 keratinocytes (µg/mL)	MIC / CC50	Selectivity index (CC50 / MIC)
Melittin	2.5	3.91	0.64	1.56
Bee venom	10	2.18	4.59	0.22
Apamin	> 10	> 10	—	—

5. Statistical analysis

Table S9. Two-way analysis of variance (Type II) for the concentration × LPS design, per compound (effect size η^2_p). Concentration was the dominant factor for bee venom and melittin ($\eta^2 \approx 0.98$). The significant LPS effect for apamin in the LDH assay ($F = 13.47$; $p = 0.002$) most likely reflects a general influence of LPS on the LDH signal rather than a compound-specific response.

Assay	Compound	Conc. F	Conc. p	η²	LPS F	LPS p	Interact. p	Shapiro / Levene p
MTT	Bee venom	1737.6	2.6e-21 ***	0.99	25.26	0.000 ***	0.000 ***	0.05 / 0.39
MTT	Melittin	533.2	9.6e-17 ***	0.98	2.31	0.146 ns	0.489 ns	0.28 / 0.25
MTT	Apamin	5.4	1.4e-02 *	0.38	0.02	0.881 ns	0.827 ns	0.77 / 0.49
LDH	Bee venom	144.5	8.2e-12 ***	0.94	5.07	0.037 *	0.024 *	0.34 / 0.45
LDH	Melittin	61.6	8.9e-09 ***	0.87	0.01	0.904 ns	0.829 ns	0.60 / 0.05
LDH	Apamin	4.0	3.5e-02 *	0.31	13.47	0.002 **	0.597 ns	0.63 / 0.84

Table S10. Pairwise comparisons of the −LPS and +LPS conditions at each concentration (Welch t-test with Holm correction; Cohen d). The only comparison surviving correction was bee venom at 5 µg/mL in the MTT assay ($p\_Holm = 0.008$, $d = -3.71$), confirming the LPS-mediated attenuation of cytotoxicity at submaximal concentration.

Assay	Compound	Conc. (µg/mL)	Mean −LPS	Mean +LPS	t	p (Welch)	p (Holm)	Cohen d	Sig.
MTT	Bee venom	1	71.1	72.1	0.305	0.611	0.611	−0.81	ns
MTT	Bee venom	5	27.5	39.2	−5.254	0.003	0.008	−3.71	**
MTT	Bee venom	10	12.3	12.1	0.200	0.784	0.784	0.20	ns
MTT	Melittin	1	86.9	90.5	−1.270	0.132	0.397	−1.27	ns
MTT	Melittin	5	43.4	47.0	−1.120	0.169	0.397	−1.12	ns
MTT	Melittin	10	29.5	29.2	0.070	0.929	0.929	0.07	ns
MTT	Apamin	1	95.1	94.1	0.240	0.756	1.000	0.24	ns
MTT	Apamin	5	87.3	88.6	−0.280	0.711	1.000	−0.28	ns
MTT	Apamin	10	90.4	89.4	0.260	0.724	1.000	0.26	ns
LDH	Bee venom	1	41.6	39.7	0.490	0.514	1.000	0.49	ns
LDH	Bee venom	5	74.1	60.5	2.420	0.017	0.051	2.42	ns
LDH	Bee venom	10	83.4	84.7	−0.230	0.763	1.000	−0.23	ns
LDH	Melittin	1	35.1	32.8	0.590	0.437	1.000	0.59	ns
LDH	Melittin	5	67.8	70.0	−0.320	0.665	1.000	−0.32	ns
LDH	Melittin	10	71.7	72.9	−0.120	0.876	1.000	−0.12	ns
LDH	Apamin	1	40.6	32.7	1.680	0.059	0.176	1.68	ns
LDH	Apamin	5	40.0	36.0	1.150	0.156	0.176	1.15	ns
LDH	Apamin	10	44.8	39.2	1.670	0.080	0.176	1.67	ns

Table S11. Multiple comparisons of each treated group versus the vehicle (DMSO) control by one-way ANOVA followed by Dunnett's test (MTT, −LPS). Bee venom was significantly cytotoxic at all concentrations; melittin and apamin were non-significant at 1 µg/mL.

Compound	ANOVA F	ANOVA p	p (1 µg/mL)	p (5 µg/mL)	p (10 µg/mL)
Bee venom	1428.9	1.4e-15	0.000 ***	0.000 ***	0.000 ***
Melittin	139.3	1.4e-09	0.709 ns	0.000 ***	0.000 ***
Apamin	7.6	4.2e-03	0.414 ns	0.002 **	0.021 *

Table S12. Spearman rank correlation between assay outcomes across the nine −LPS conditions (three compounds × three concentrations). Loss of viability was associated with increased LDH release and with apoptosis, indicating a consistent direction of the cellular responses.

Pair	Spearman ρ	t	p (two-sided)	Interpretation
Viability (MTT) vs LDH	−0.80	−3.53	0.010	inverse, strong
Apoptosis vs LDH	+0.75	2.99	0.020	positive, strong

Pair	Spearman ρ	t	p (two-sided)	Interpretation
Viability (MTT) vs Apoptosis	−0.58	−1.88	0.099	not significant

6. Raw per-well data

Table S13. Individual replicate-well values (percentage of control) underlying the MTT and LDH summary statistics (Tables S2–S3). Values represent the four technical wells per condition from a single experimental passage.

Assay	Compound	Conc. (µg/mL)	LPS	Well 1	Well 2	Well 3	Well 4
MTT	Bee venom	1	−LPS	69.9	71.1	71.9	71.5
MTT	Bee venom	1	+LPS	74.4	71.8	71.5	70.8
MTT	Bee venom	5	−LPS	22.1	29.7	29.9	28.4
MTT	Bee venom	5	+LPS	41.7	41.0	38.0	36.3
MTT	Bee venom	10	−LPS	11.2	12.0	12.7	13.5
MTT	Bee venom	10	+LPS	13.3	12.3	11.9	11.0
MTT	Melittin	1	−LPS	86.5	86.9	82.8	91.3
MTT	Melittin	1	+LPS	91.8	87.7	92.4	90.2
MTT	Melittin	5	−LPS	38.6	44.4	43.0	47.5
MTT	Melittin	5	+LPS	50.5	46.1	47.5	44.0
MTT	Melittin	10	−LPS	25.8	23.8	34.9	33.7
MTT	Melittin	10	+LPS	28.5	33.4	31.0	23.9
MTT	Apamin	1	−LPS	95.2	96.5	92.9	95.8
MTT	Apamin	1	+LPS	100.1	96.6	88.0	91.9
MTT	Apamin	5	−LPS	84.7	86.9	83.3	94.5
MTT	Apamin	5	+LPS	87.5	84.7	93.8	88.3
MTT	Apamin	10	−LPS	88.8	84.6	93.7	94.7
MTT	Apamin	10	+LPS	85.7	87.8	93.5	90.5
LDH	Bee venom	1	−LPS	40.0	36.7	43.0	46.9
LDH	Bee venom	1	+LPS	35.9	38.2	42.4	42.4
LDH	Bee venom	5	−LPS	78.1	75.3	67.6	75.4
LDH	Bee venom	5	+LPS	53.8	69.5	59.6	59.1
LDH	Bee venom	10	−LPS	78.1	84.7	87.0	83.7
LDH	Bee venom	10	+LPS	90.8	91.1	78.7	78.2
LDH	Melittin	1	−LPS	34.3	30.7	40.8	34.5
LDH	Melittin	1	+LPS	33.2	35.5	34.9	27.7
LDH	Melittin	5	−LPS	61.8	65.3	73.1	70.9
LDH	Melittin	5	+LPS	61.1	68.7	69.6	80.4
LDH	Melittin	10	−LPS	61.8	81.7	71.4	71.8
LDH	Melittin	10	+LPS	63.6	85.0	82.9	60.2
LDH	Apamin	1	−LPS	48.4	39.7	36.0	38.5
LDH	Apamin	1	+LPS	27.2	35.8	35.6	32.0
LDH	Apamin	5	−LPS	36.7	42.9	43.1	37.3
LDH	Apamin	5	+LPS	34.1	33.2	35.8	40.9
LDH	Apamin	10	−LPS	39.6	43.4	46.3	50.1
LDH	Apamin	10	+LPS	37.6	40.3	40.9	37.9

Supplementary Figures

The following figures present the analytical chromatograms and mass spectra, the fluorescent cell-death micrographs, and the representative microbiological plates supporting the results summarized above.

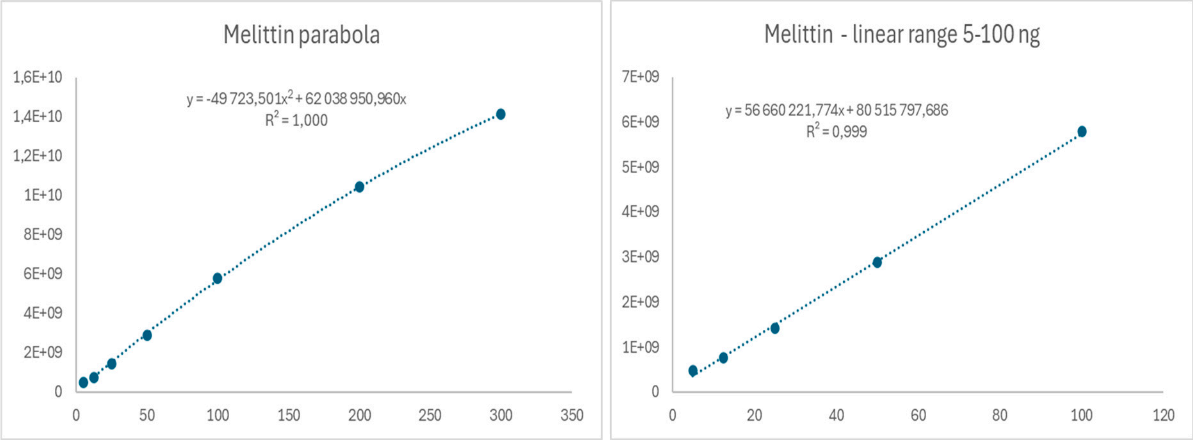

Figure S1. The calibration curve for the quantification of melittin in bee venom

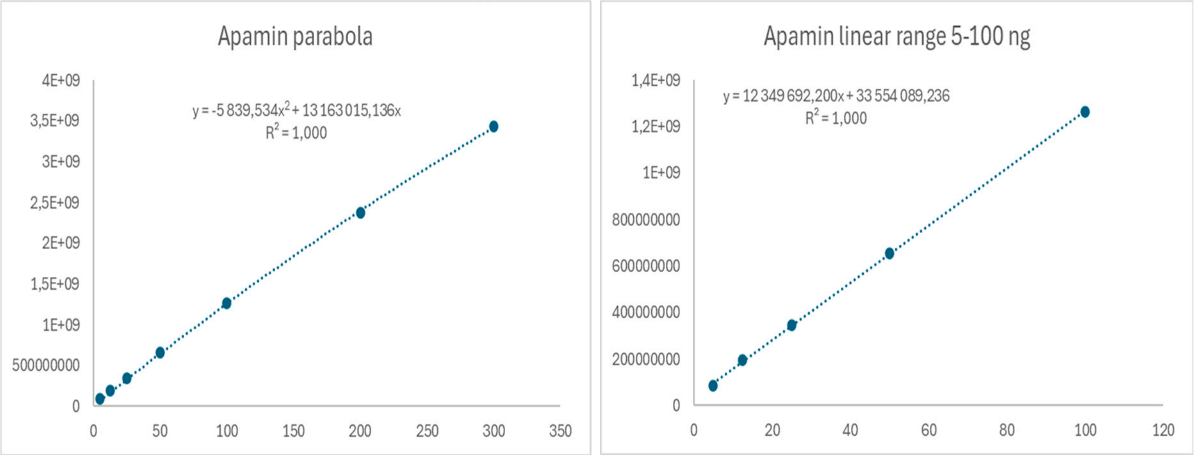

Figure S2. The calibration curve for the quantification of apamin in bee venom

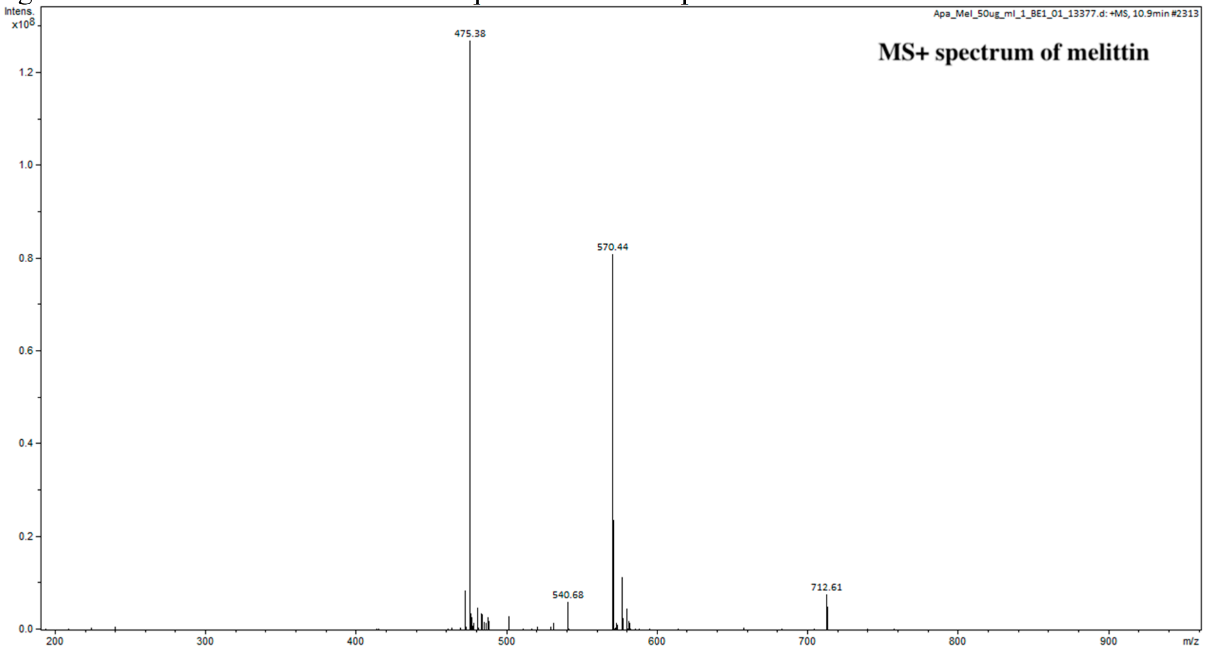

Figure S3. Positive ion electrospray mass spectrum (MS+) of melittin acquired by UHPLC–ESI–MS. The dominant ions detected at m/z 475.38, 570.44, and 712.61 correspond to the multiply protonated forms of melittin

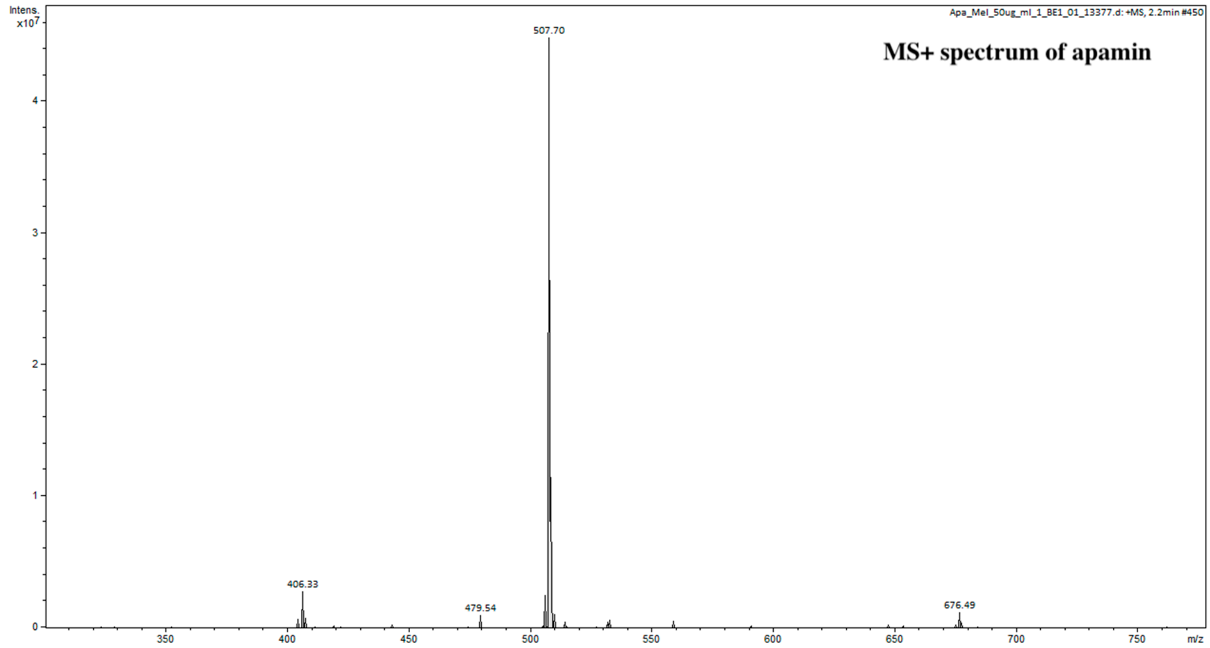

Figure S4. Positive ion electrospray mass spectrum (MS+) of apamin acquired by UHPLC–ESI–MS. The dominant ion detected at m/z 507.70 corresponds to the protonated molecular ion of apamin

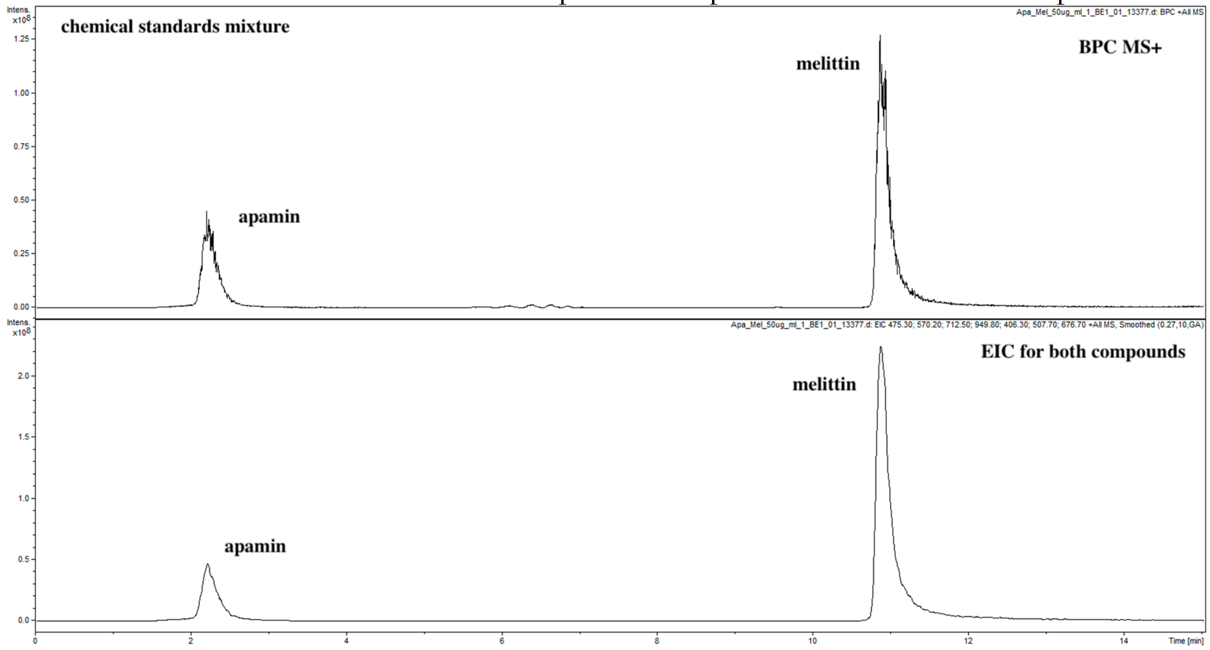

Figure S5. UHPLC–ESI–MS chromatograms of the mixture of apamin and melittin chemical standards recorded in positive ion mode. The upper panel shows the base peak chromatogram (BPC MS+) and the lower panel the extracted ion chromatogram (EIC MS+) for both compounds.

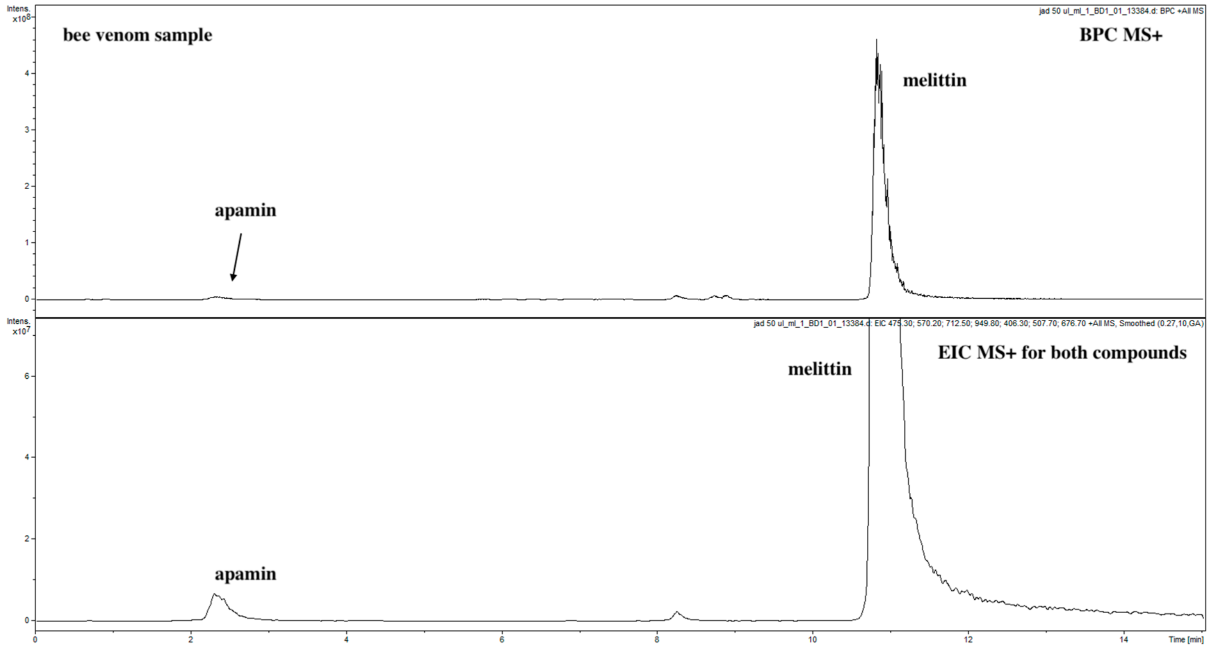

Figure S6. UHPLC–ESI–MS chromatograms of the bee venom sample (BPC and EIC). The upper panel shows the base peak chromatogram (BPC MS+), while the lower panel presents the extracted ion chromatogram (EIC MS+) for apamin and melittin, confirming their retention times and relative abundance in the analyzed sample.

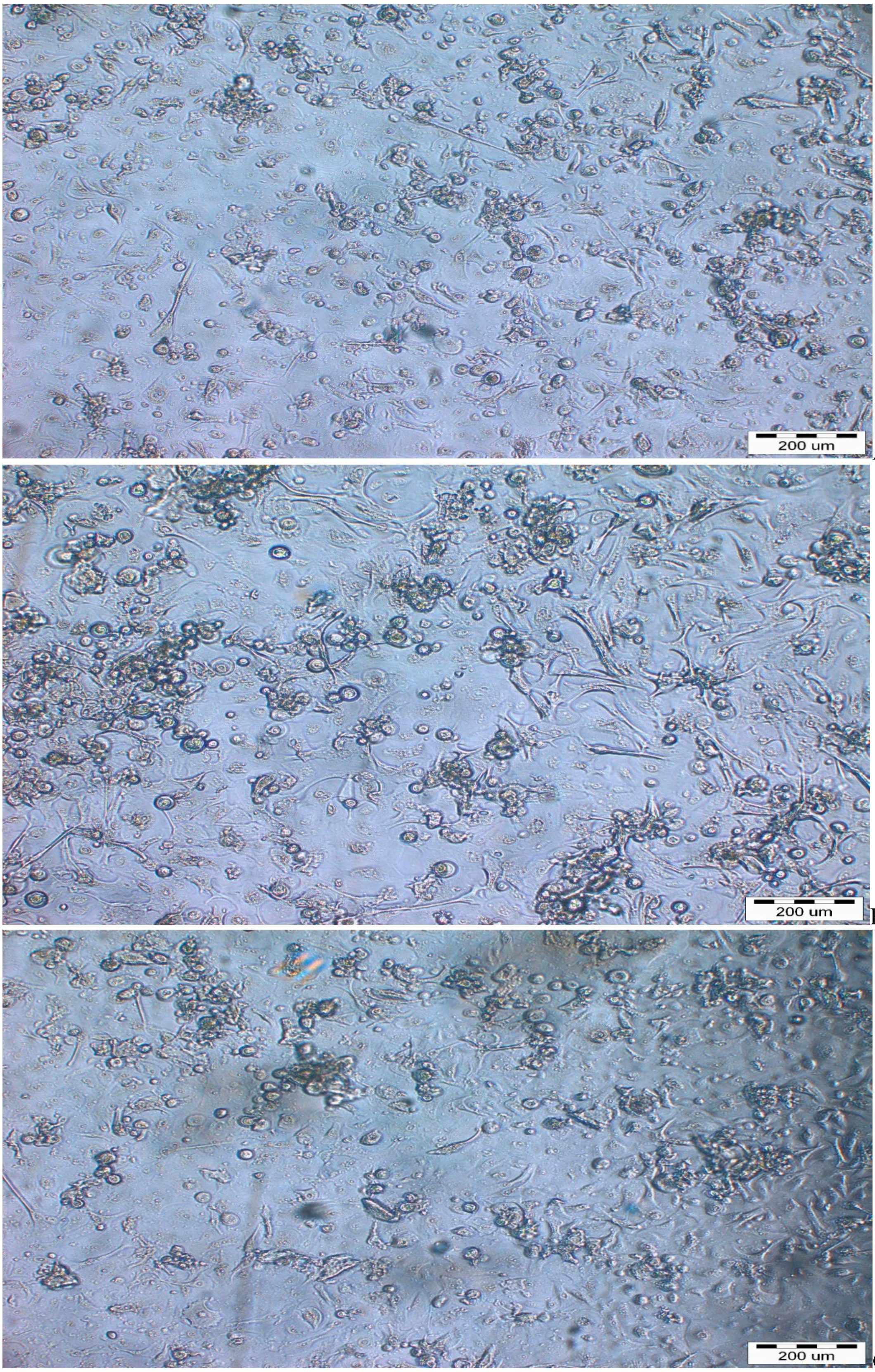

A
B
C

Figure S7. HEKa cells following stimulation with bee venom (BV) at different concentrations: (A) 1 µg/mL, (B) 5 µg/mL, and (C) 10 µg/mL

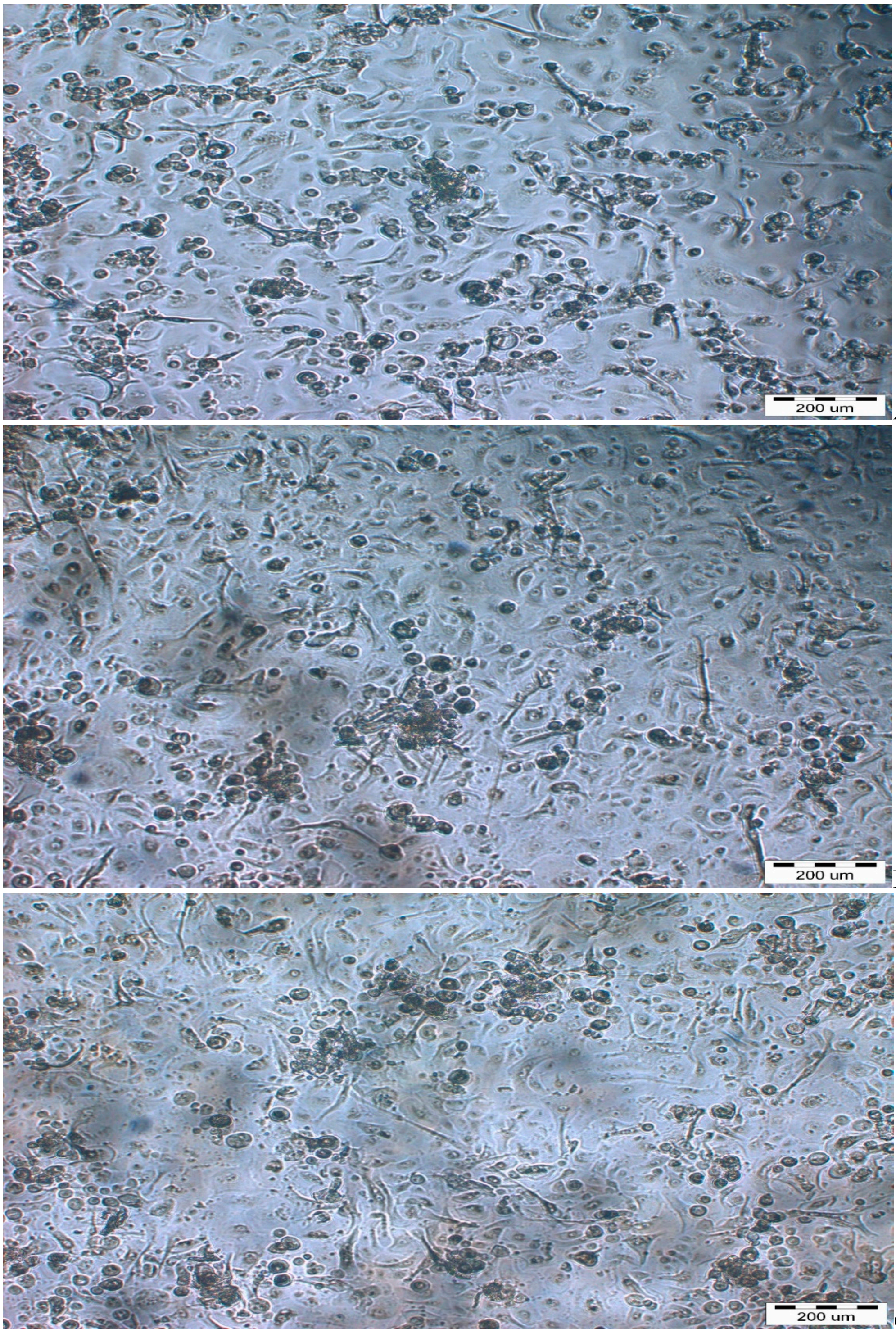

A
B
C

Figure S8. HEKa cells following stimulation with melittin (MEL) at different concentrations: (A) 1 µg/mL, (B) 5 µg/mL, and (C) 10 µg/mL

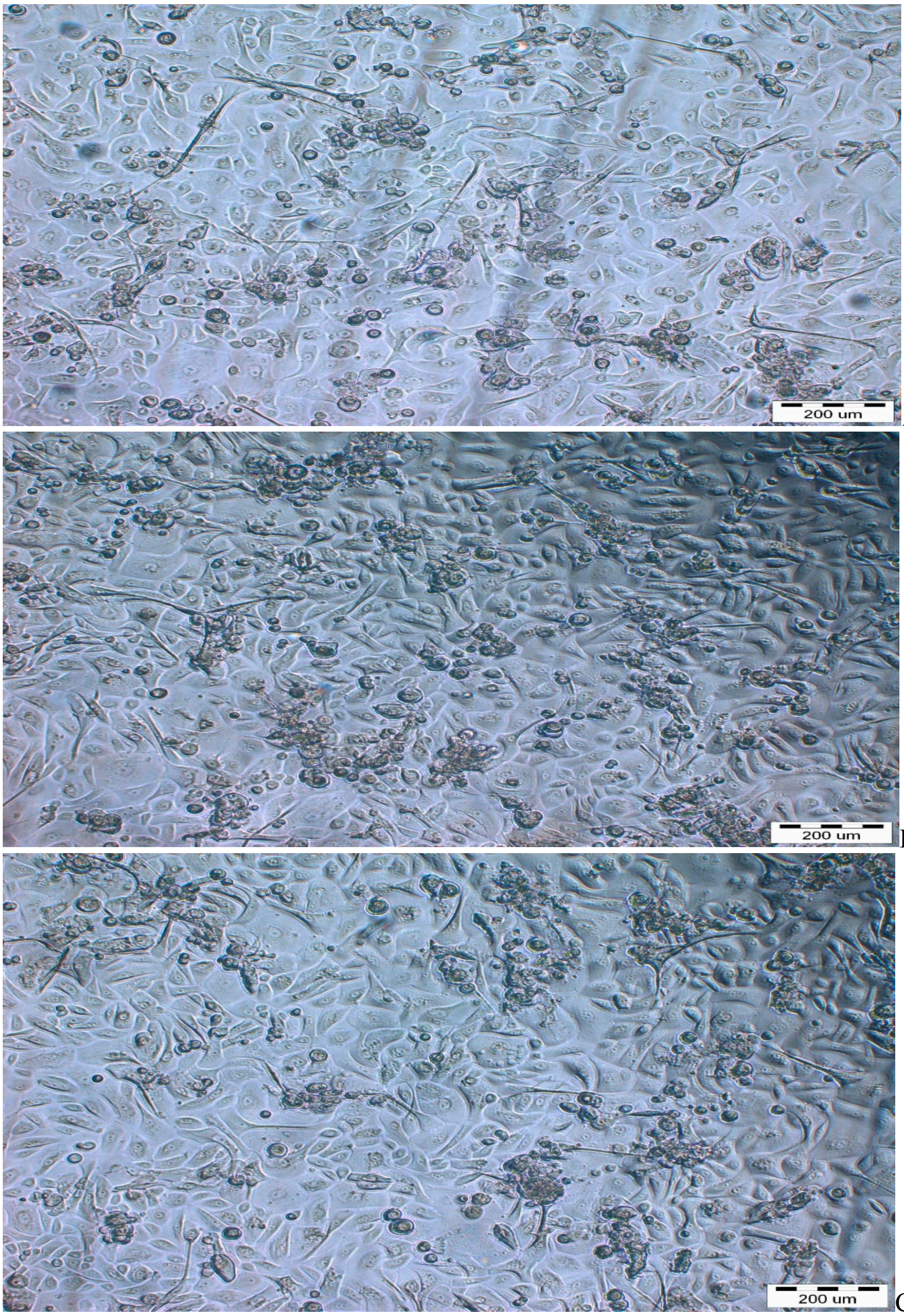

A B C

Figure S9. HEKa cells following stimulation with apamin (APA) at different concentrations: (A) 1 µg/mL, (B) 5 µg/mL, and (C) 10 µg/mL

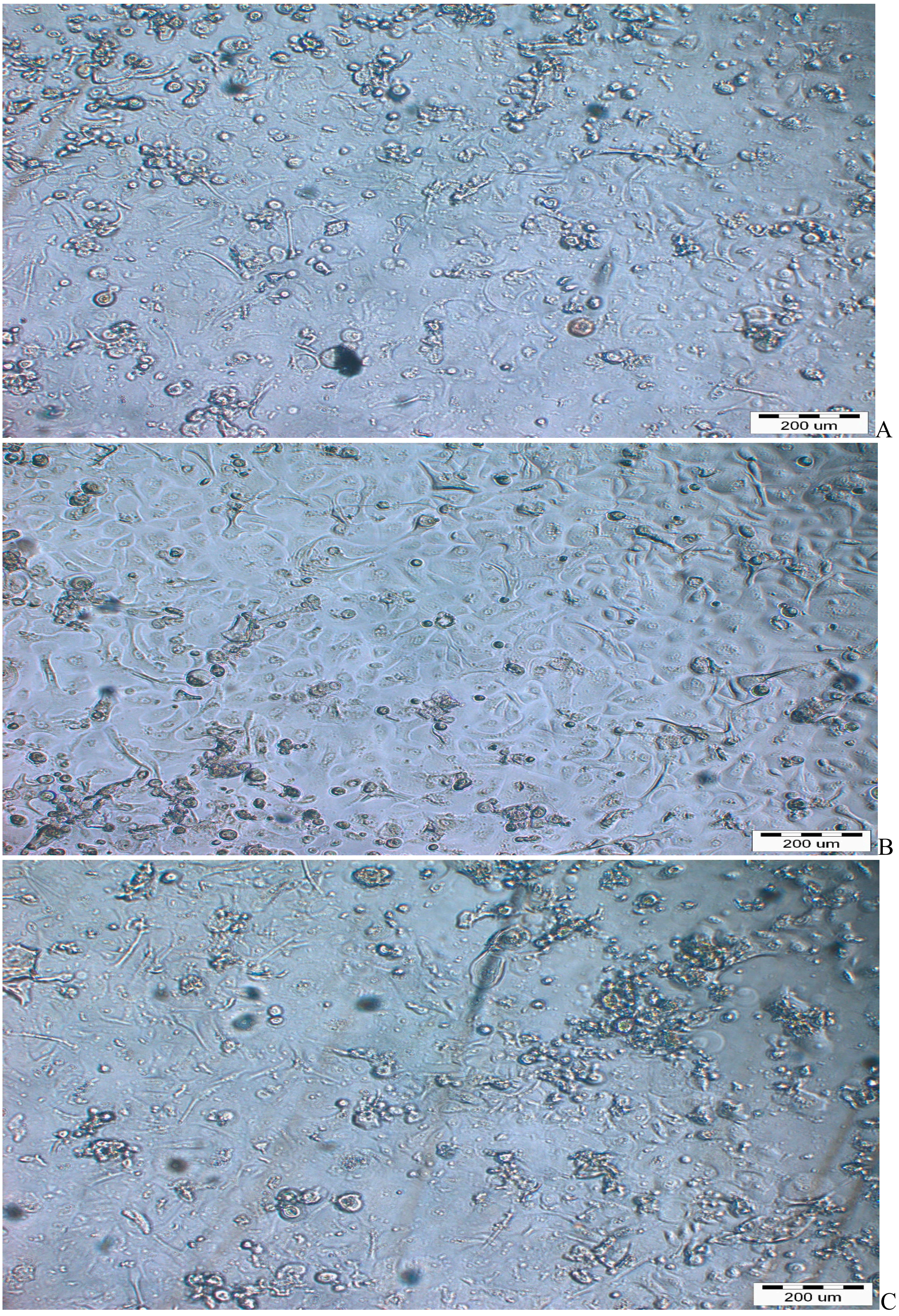

Figure S10. HEKa cells following stimulation with camptothecin

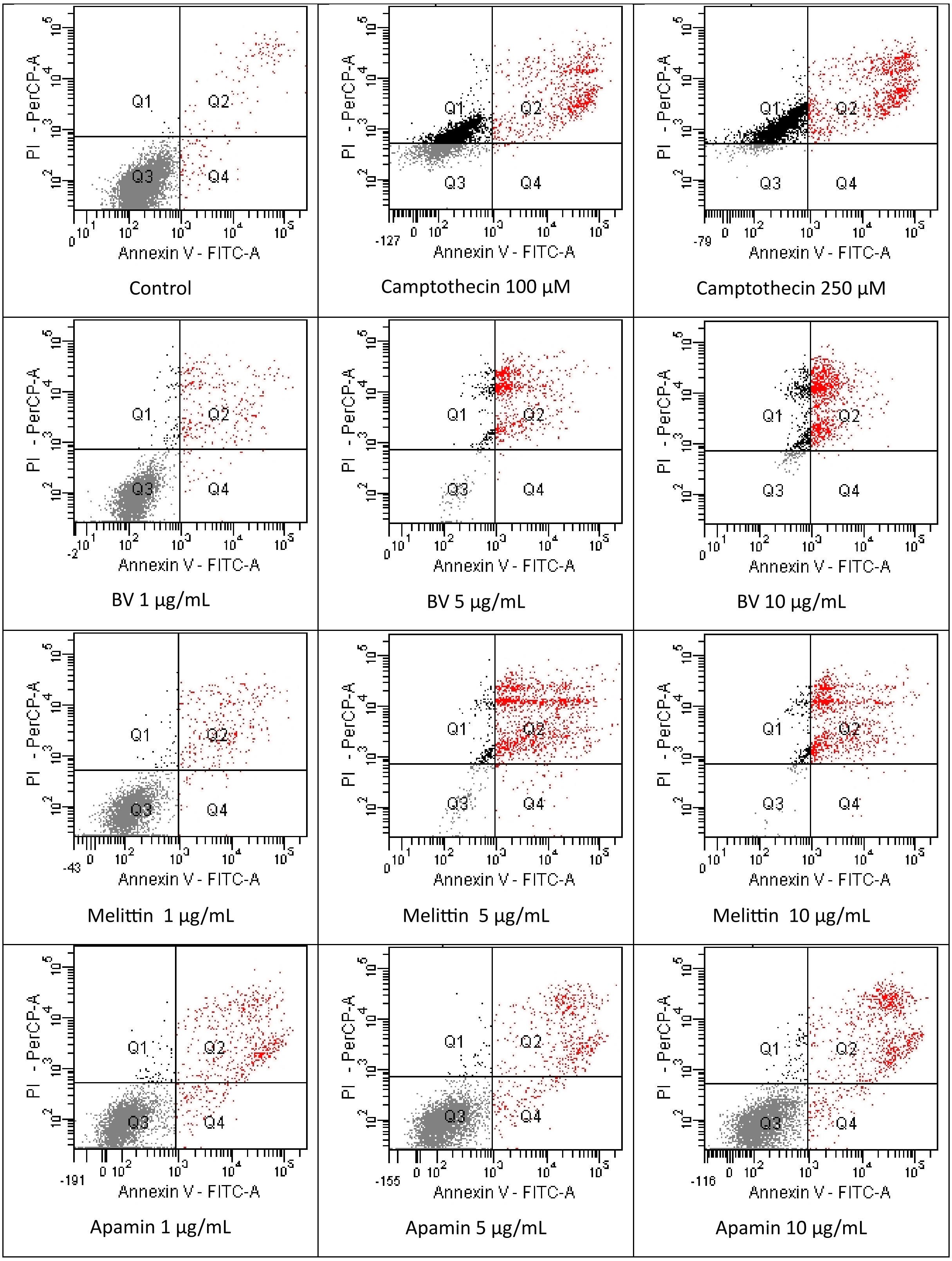

Figure S11. Apoptotic effect (showed as dot plots) of BV, melittin and apamin (1, 5, 10 µg/mL) in HEKa cells. Camptothecin (100, 250 µM) was used as positive control. Q1 – dead cells, Q2 – late apoptosis, Q3 – early apoptosis, Q4 – non-apoptotic cells.

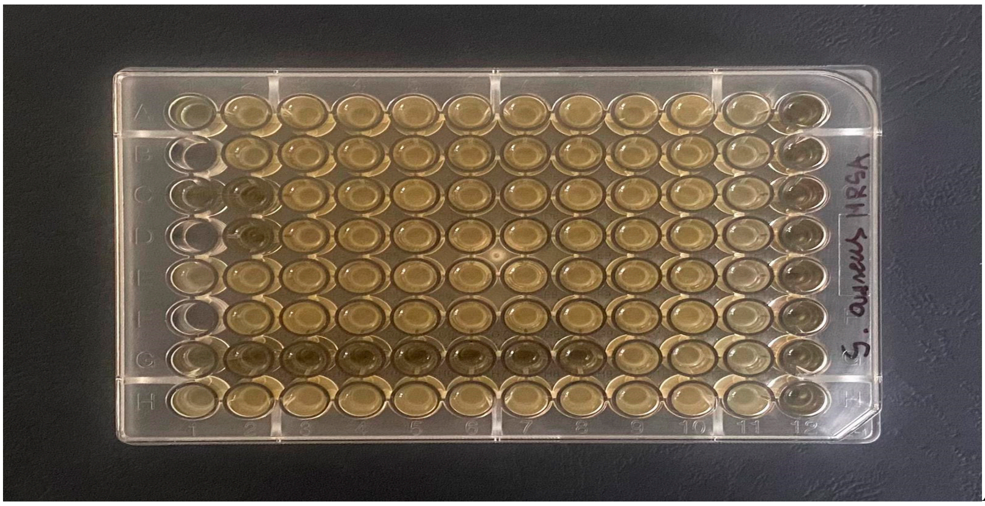

A

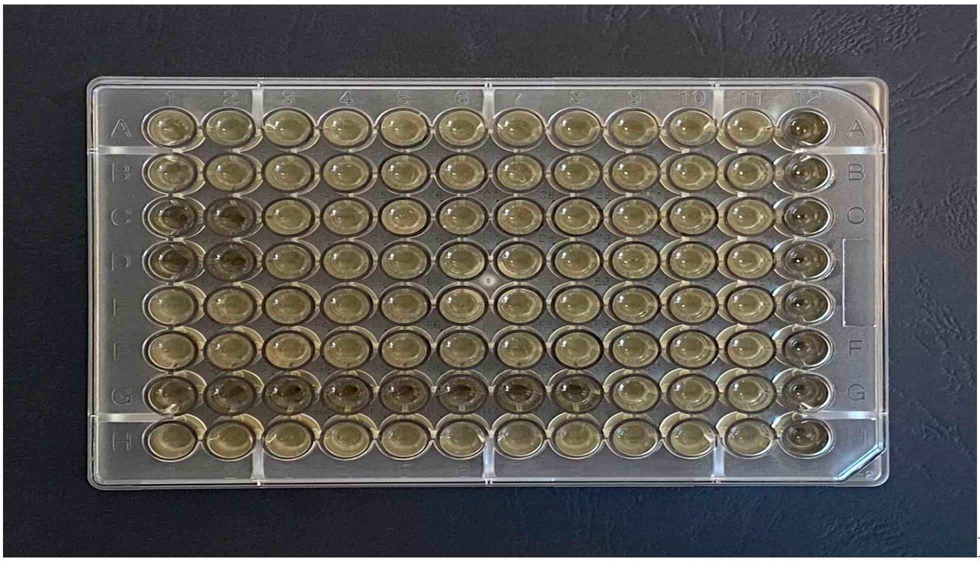

B

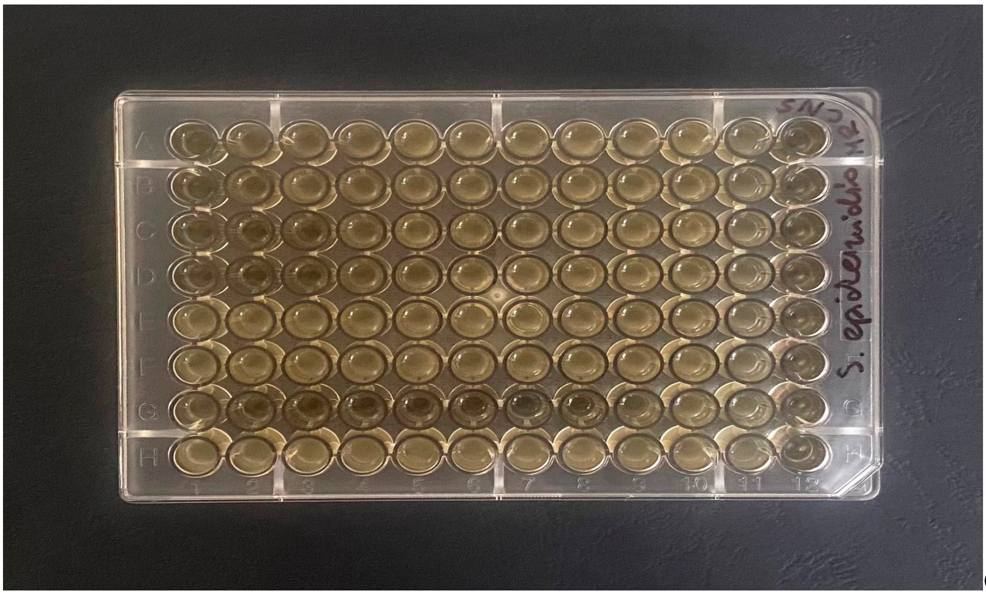

C

Figure S12. Representative 96-well microtiter plate used for determination of the minimum inhibitory concentration (MIC) of the tested compounds against *Staphylococcus aureus* MRSA - (A), *Staphylococcus aureus* MSSA - (B), *Staphylococcus epidermidis* MRSE - (C) using the broth microdilution method.

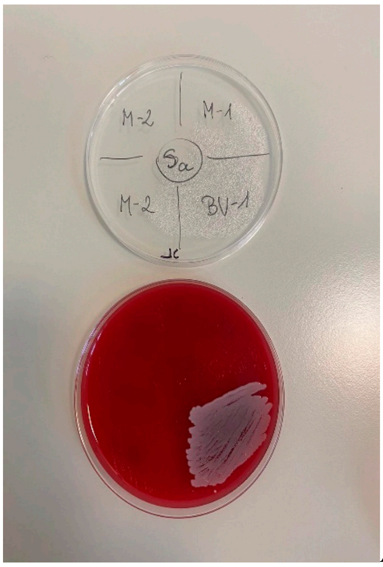

A

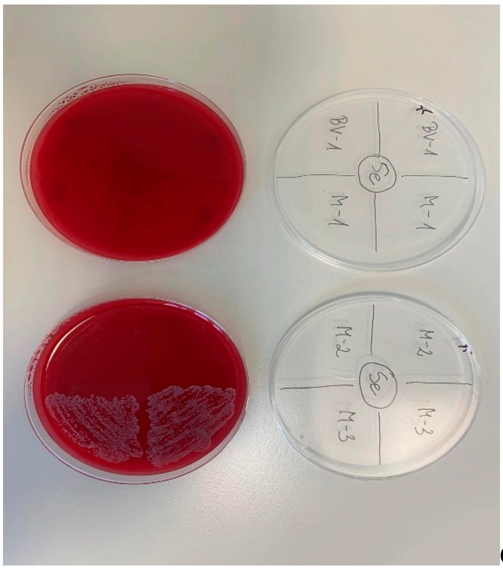

C

Figure S13. Representative blood agar plates illustrating the assessment of minimum bactericidal concentration (MBC) for bee venom (BV) and melittin against *Staphylococcus aureus* MRSA - (A), *Staphylococcus aureus* MSSA - (B), *Staphylococcus epidermidis* MRSE - (C). MBC values were determined by subculturing samples from MIC assay wells onto blood agar plates and evaluating bacterial growth after incubation

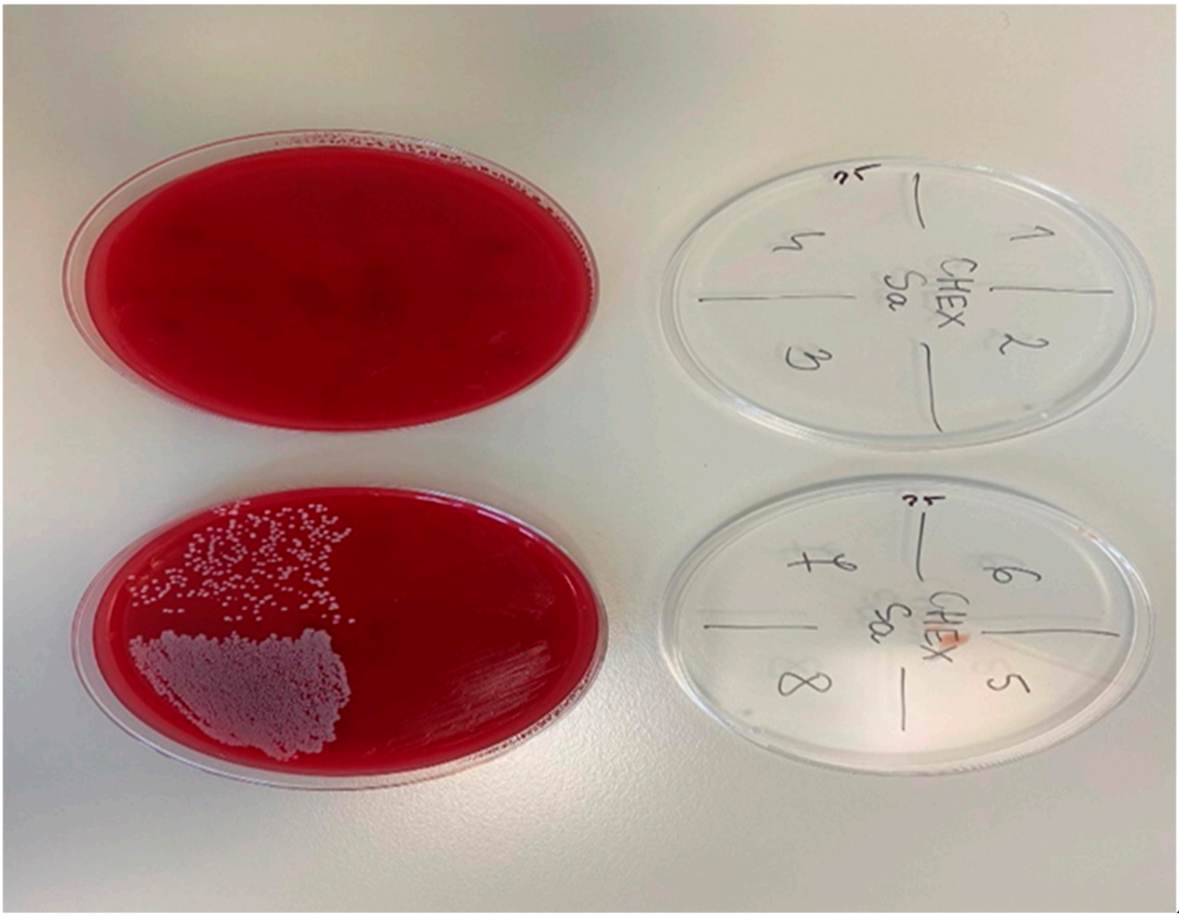

A

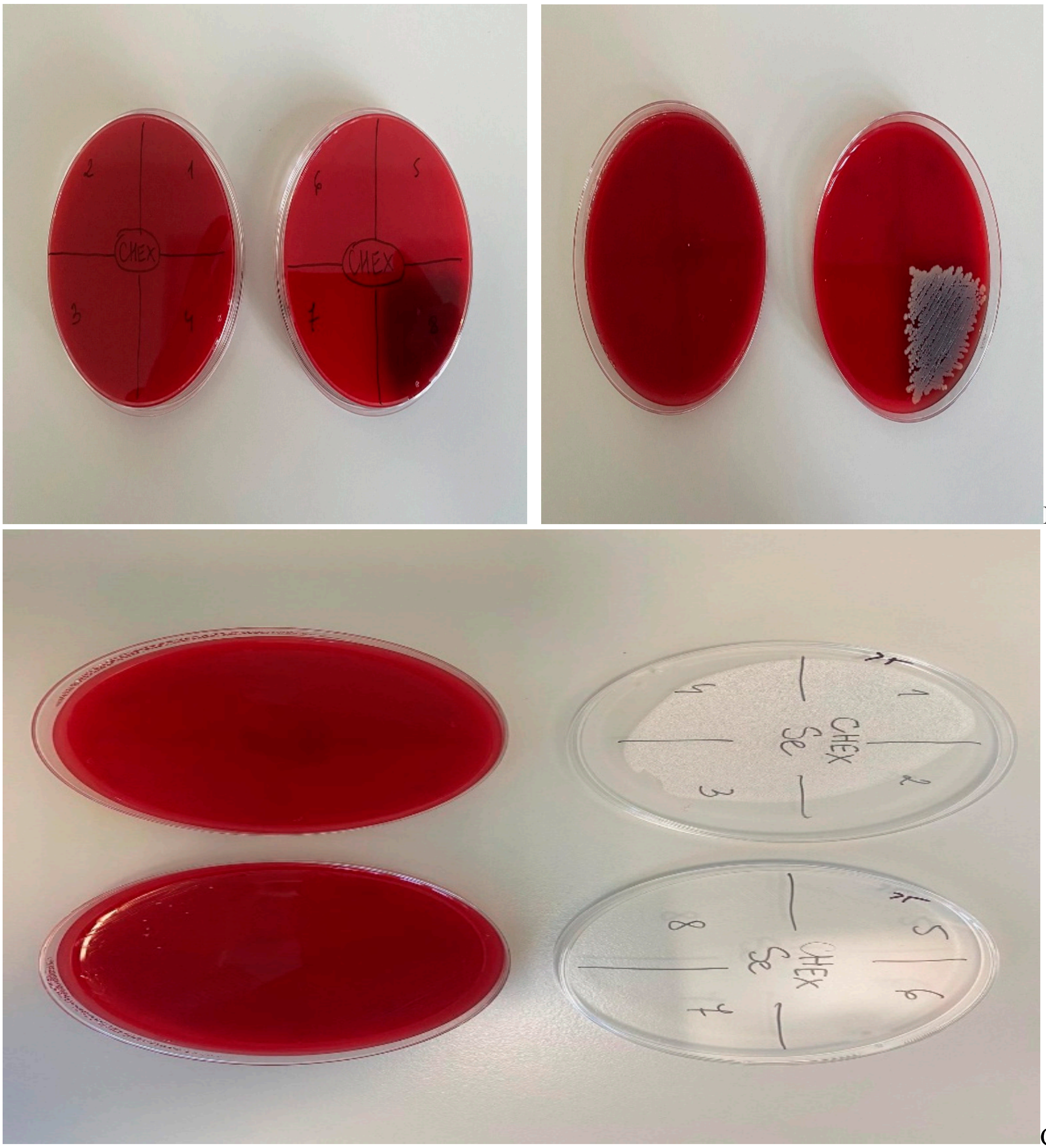

B

C

Figure S14. Representative blood agar plates illustrating the assessment of minimum bactericidal concentration (MBC) for chlorhexidine(CHX) against *Staphylococcus aureus* MRSA - (A), *Staphylococcus aureus* MSSA - (B), *Staphylococcus epidermidis* MRSE - (C). MBC values were determined by subculturing samples from MIC assay wells onto blood agar plates and evaluating bacterial growth after incubation
